# Proteome Remodeling of a Carbapenem-Resistant *Escherichia coli* Strain upon Meropenem Exposure

**DOI:** 10.1021/acs.jproteome.6c00342

**Published:** 2026-07-14

**Authors:** Leonarda Acha Alarcon, Daniel Jaén-Luchoro, Francisco Salvà-Serra, Edward R. B. Moore, Ivan Mijakovic, Roger Karlsson

**Affiliations:** † Department of Infectious Diseases, Institute for Biomedicine, Sahlgrenska Academy of the University of Gothenburg, Gothenburg 41346, Sweden; ‡ Centre for Antibiotic Resistance Research (CARe), 378993 University of Gothenburg, Gothenburg 41346, Sweden; § Department of Clinical Microbiology, 56749 Sahlgrenska University Hospital, Region Västra Götaland, Gothenburg 41346, Sweden; ∥ Culture Collection University of Gothenburg (CCUG), Sahlgrenska Academy of the University of Gothenburg, Gothenburg 41346, Sweden; ⊥ Unit of Medical Technology and Biological Function, Department of Medical Technology and Diagnostics, RISE Research Institutes of Sweden, Gothenburg 43153, Sweden; # Systems Biology, Life Sciences, Chalmers University of Technology, Gothenburg 41296, Sweden; ¶ Nanoxis Consulting AB, Gothenburg 40016, Sweden

**Keywords:** *Escherichia coli*, carbapenem resistance, mass spectrometry, quantitative proteomics

## Abstract

Carbapenem-resistant *Escherichia coli* poses a serious threat to global health, with limited treatment options and potentially fatal outcomes. Beyond horizontally acquired resistance genes, other cellular pathways are remodeled in the resistant phenotype, but these systemic responses remain poorly understood. Here, tandem mass tag-based quantitative proteomics was used to characterize the response of *E. coli* strain CCUG 70745 to Meropenem. Two inhibitory concentrations (128 and 192 μg/mL), anchored to the MIC of the isolate (128 μg/mL), were compared with an antibiotic-free control after 15 min. The analysis quantified 60% of the theoretical proteome, identifying 172 and 813 differentially expressed proteins at 128 and 192 μg/mL, respectively. Canonical resistance determinants, including β-lactamases and efflux pumps, were altered, as well as proteins involved in cell–wall formation, membrane transport, and signal transduction. Gene set enrichment analysis highlighted oxidative phosphorylation, ABC transporters, two-component systems, and cofactor metabolism. Increased abundances of two-component-system and membrane-integrity proteins were consistent with a coordinated, dose-dependent stress response, and interaction networks linked a cell-division module to efflux and metabolic adaptation. These results suggest that Meropenem engages both primary and auxiliary resistance-associated processes, providing a proteomic framework that may guide therapies targeting bacterial metabolism and membrane functions.

## Graphical abstract

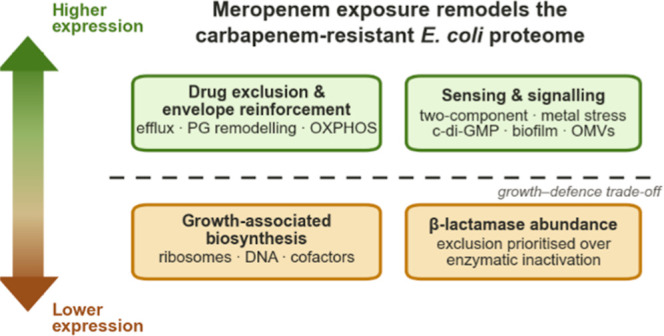

## Introduction

According to the latest Global Burden of Disease report from the Antimicrobial Resistance Collaborators, antibiotic-resistant bacteria caused 1.14 million deaths in 2021. The same report, aligned with previous predictions for 2050, forecasts an increase in mortality.^1^ Antimicrobial resistance (AMR) results from bacterial adaptation to antibiotic pressure; therefore, antibiotics at clinical concentrations are ineffective against bacteria. The effectiveness of antibiotics depends on the bacteria’s metabolic state, and the acquisition of resistance will impact the bacterial fitness.^2^ Common AMR mechanisms include target-site modifications, reduced influx, increased efflux, enzymatic inactivation of antibiotics, and alterations in metabolic pathways.^3^ Four main metabolic processes contribute to AMR: cell envelope biogenesis; DNA replication; transcription; and protein biosynthesis.^2^ Evolutionary studies highlight antibiotic resistance arising from gene mutations that induce global transcriptomic changes affecting thousands of genes involved in oxidative stress regulation, iron homeostasis, and outer membrane and cell-surface composition.^4^

Carbapenems, a class of β-lactam antibiotics, are last-resort antibiotics, used to treat serious infections caused by resistant bacteria or when previous antibiotic treatments have failed.^5^ During the past 30 years, there has been a concerning 70% increase in carbapenem resistance among Gram-negative bacteria. Since 1990, the number of deaths attributable to AMR and *Escherichia coli* has been rising (11.1%–14%),^1^ and in 2019, *E. coli* was reported as the leading pathogen responsible for most AMR-related deaths.^6^ Furthermore, *E. coli* is among the leading pathogens accountable for the highest disease burden worldwide.^7^

Carbapenem-resistant *E. coli* (CREC) strains primarily exhibit two resistance mechanisms to carbapenems: decreased permeability due to porin loss and the production of hydrolytic enzymes, including extended-spectrum β-lactamases and carbapenemases (e.g., blaOXA-48 and blaNDM).^8,9^ A profound rewiring of metabolism that affects bacterial morphology has been described in CREC, whose resistance mechanism depends on porins.^10^ Furthermore, other approaches reported that cell division, cell envelope synthesis and maintenance, ATP metabolism, and transcriptional regulation were affected by Meropenem.^11^ Given the complexity of adaptations that occur upon exposure to carbapenems, a holistic approach is necessary to understand these metabolic changes within the broader cellular context.

The link between *E. coli* and resistance to last-resort antibiotics poses serious health risks; therefore, it is essential to develop strategies to prevent deaths caused by Gram-negative bacteria and antimicrobial resistance (AMR). Studying AMR from a broader perspective could reveal new or additional targets that could slow the rapid rise of AMR. Quantitative proteomics approaches are particularly suitable for this purpose because they offer a more comprehensive view of the expressed proteins, that is, the actual effectors that confer the resistant phenotype.

The genome of the *E. coli* strain CCUG 70745 (ST-648), isolated from a human fecal sample at Sahlgrenska University Hospital (Gothenburg, Sweden) in 2013, was previously characterized and found to carry multiple resistance genes.^12^ Here, a quantitative proteomics approach, using bottom-up mass spectrometry, was employed to examine differential expression in strain CCUG 70745 under Meropenem treatment. To achieve this, bioinformatic analyses were conducted, including protein–protein interaction networks, functional annotations, and enrichment analyses.

Previous proteomics studies have examined bacterial responses to antibiotics and resistance mechanisms, but most have focused on longer exposure times, susceptible strains, or broad stress conditions and often emphasized descriptive protein lists rather than integrated network-level interpretations. In contrast, the present study combines short-term (15 min) TMT-based quantitative proteomics with clustering, gene set enrichment, and protein–protein interaction analyses to resolve the very early, concentration-dependent response of a carbapenem-resistant *E. coli* clinical isolate to Meropenem. This design allows us to link canonical resistance determinants to coordinated changes in cell-envelope remodeling, transport and ion-homeostasis systems, signaling (including two-component and c-di-GMP pathways), and respiratory and metabolic reprogramming. By providing a systems-level view of how these modules are rapidly engaged upon carbapenem exposure, our work extends previous antibiotic-response proteomics and offers a framework for identifying auxiliary processes that may be exploited in future therapeutic strategies.

## Experimental Procedures

The multidrug-resistant *E. coli* strain CCUG 70745 was used in this study. The strain was obtained from the Culture Collection University of Gothenburg (CCUG), grown for 18 h on a Columbia Blood Agar Base plus 5% defibrinated horse blood agar medium plate at 37 °C, and its genetic background was confirmed by whole-genome sequencing as a control prior to performing the proteomics experiments (Supporting Information 1).

### Antibiotic Susceptibility Testing

A susceptibility test to confirm the antibiotic susceptibility profile, described elsewhere,^12^ was conducted at the National Reference Laboratory for Antibiotic Resistance in Sweden (Växjö, Sweden; http://www.mikrobiologi.org/referenslaboratorium). The test determined the minimum inhibitory concentration (MIC) using the broth dilution method with the “*Enterobacteriales* standard panel,” following the European Committee on Antimicrobial Susceptibility Testing (EUCAST) recommendations and the ISO 20776-1 (2006) standard. To determine the antibiotic concentrations used in the proteomics experiments, the MIC of Meropenem was measured in-house using the same method. Briefly, a bacterial suspension with 1.77 × 10^5^ CFU/mL was used to inoculate 96-well microplates containing cation-adjusted Mueller Hinton broth (Substrate Unit, Department of Clinical Microbiology, Sahlgrenska University Hospital; GBG; SE) with 2-fold serial dilutions of Meropenem trihydrate (ref. 462810050; Thermo Fisher Scientific; NJ; USA) ranging from 4 μg/mL to 2048 μg/mL. The inoculated plates were incubated in the Multiskan FC (ref. 51119000; Thermo Fisher Scientific; BLW; NL) instrument at 37 °C in aerobic conditions with agitation for 24 h, and the optical density (OD) at 600 nm was measured every 15 min. The MIC results were interpreted using the EUCAST MIC breakpoints v. 14.0 (2024; www.eucast.org/clinicalbreakpoints), following the broth dilution method and the “*Enterobacteriales* standard panel” of ISO 20776-1 (2006).

### Bacterial Growth and Testing of the Antibiotic Impact on the Strain CCUG 70745

To select the appropriate time point for bacterial growth and to challenge the culture with Meropenem, growth curves were determined from three isolated colonies. A preinoculum of the strain was incubated for 18 h at 37 °C under agitation (180 rpm) in 5 mL of cation-adjusted Mueller–Hinton broth; from there, an aliquot of 200 μL was used to seed 100 mL of cation-adjusted Mueller–Hinton, the flasks were incubated in aerobic conditions at 37 °C under agitation (180 rpm), and OD measurements at a wavelength of 600 nm were taken in a CO8000 Cell Density Meter (ref. 490005-906; WPA Biowave; CB; UK) every 30 min for a total of 510 min.

Once the growth phases of CCUG 70745 were identified, the effect of Meropenem was evaluated during the midexponential phase. Therefore, 100 mL of cation-adjusted Mueller–Hinton broth was inoculated with approximately 1.9 × 10^8^ CFU (200 μL) of a 12 h preinoculum and incubated under the same conditions as previously described for 2 h (OD_600_ > 0.15). Different concentrations of Meropenem, ranging from 32 μg/mL to 256 μg/mL, were added to each flask. The antibiotic’s effect was assessed by measuring changes in OD600 over 8.5 h. Growth curves, based on time and optical density, were created and compared to select the antibiotic concentrations for the proteomics experiment.

### Proteomic Sample Preparation for Quantitative Proteomics

For the proteomics experiment, 100 mL of cation-adjusted Mueller–Hinton broth was inoculated as described in the previous section. After 2 h of incubation, two different concentrations of Meropenem (128 μg/mL and 192 μg/mL) were added to separate cultures, exposing the bacteria to the antibiotic for 15 min. The samples were obtained from five biological replicates. Cultures without antibiotics served as controls. The biomass from the controls was collected at two time points: 2 h after incubation and 15 min after mock control addition (Mueller Hinton without antibiotics) from two separate flask sets. Four biological replicates for each control were collected for analysis. The biomass was harvested by centrifugation at 5,000*g* for 15 min at 4 °C. The collected pellets were rinsed once with 1 mL of phosphate-buffered saline (PBS; 0.01 M; Substrate Unit, Department of Clinical Microbiology, Sahlgrenska University Hospital, Gothenburg, Sweden), and the OD at 600 nm (OD600) was adjusted to 1.5. The suspensions were centrifuged again, and the pellets were stored at −20 °C. Consecutively, cell lysates were prepared by adding 50 mM triethylammonium bicarbonate (TEAB; ref. 90114; Thermo Fisher Scientific; IL; USA) and 4% SDS (Substrate Unit, Department of Clinical Microbiology, Sahlgrenska University Hospital, GBG; SE) to a final volume of 100 μL, and the protein extracts were submitted to the Proteomics Core Facility (University of Gothenburg; GBG; SE) for liquid chromatography tandem mass spectrometry (LC–MS/MS) analyses.

### Peptide Generation and TMT Labeling

Samples (80 μg) were processed using a modified SP3 method.^13^ In brief, proteins were reduced in 10 mM dithiothreitol (DTT; ref. D1532; Thermo Fisher Scientific; CA; USA) at 60 °C for 30 min and alkylated in 20 mM iodoacetamide (ref. I10189; Thermo Fisher Scientific; CA; USA) at room temperature for 30 min. Washed hydrophobic and hydrophilic Sera-Mag SpeedBeads (Carboxylate-Modified; ref. 65152105050350; Cytiva; MA; USA) were added to the samples at a bead-to-protein ratio of 10:1. Proteins were precipitated on the beads with ethanol (final concentration 70%; ref. E7023; Sigma-Aldrich; SOL; SE), washed twice with 70% ethanol, then washed with 100% acetonitrile (ref. 34851; Sigma-Aldrich; SOL; SE), and dried at room temperature. The beads were resuspended in 50 mM TEAB, and proteins were digested with a Trypsin/Lys-C mix (1:25; ref. V5071; Promega; WI; USA) for 2 h, followed by trypsin digestion (1:50; ref. V5111; Promega; WI; USA) overnight. The magnetic beads were removed, and samples were labeled using TMTpro 18-plex isobaric mass tagging reagents (ref. A52045; Lot. XI346567 and XJ346678; Thermo Fisher Scientific; CA; USA). The labeled samples were pooled and purified using HiPPR Detergent Removal Resin and Pierce peptide desalting spin columns (ref. 88305X; Thermo Fisher Scientific; IL; USA), following the manufacturer’s instructions. The TMT set was fractionated by basic reversed-phase chromatography on a Dionex Ultimate 3000 UHPLC system (Thermo Fisher Scientific, MA, USA). Peptide separations were performed using a reversed-phase XBridge BEH C18 column (3.5 μm, 2.1 × 250 mm; Waters Corporation; MA; USA) with a stepped gradient from 3% to 80% solvent B over 90 min at a flow rate of 200 μL/min. Solvent A consisted of 25 mM ammonia (ref. 499145; Sigma-Aldrich; SOL; SE), while solvent B was 85% acetonitrile. Fractions (96) were collected from 20 to 74 min and combined into 36 final fractions. These fractions were evaporated and reconstituted in 3% acetonitrile, 0.1% trifluoroacetic acid (ref. 91707; Sigma-Aldrich; SOL; SE), and 0.015% dodecyl-β-d-maltoside (ref. D4641; Sigma-Aldrich; SOL; SE) for LC–MS3 analysis.

### LC–MS/MS and Proteomic Data Analyses

The fractions were analyzed using an Orbitrap Lumos Tribrid mass spectrometer (Thermo Fisher Scientific; MA; USA) equipped with a FAIMS Pro ion mobility system (Thermo Fisher Scientific; MA; USA) and connected to an Easy-nLC1200 liquid chromatography system (Thermo Fisher Scientific; MA; USA). Peptides were captured on an Acclaim pepmap 100 C18 trap column (100 μm × 2 cm; particle size 5 μm; Thermo Fisher Scientific; MA; USA) and separated on a custom-packed analytical column (35 cm × 75 μm; particle size 3 μm; Reprosil-Pur C18; Dr. Maisch; Ammerbuch; DE) using a stepped gradient from 5% to 35% B over 77 min at a flow rate of 300 nL per minute (Solvent A 0.2% formic acid, Solvent B 80% ACN with 0.2% formic acid). The FAIMS Pro system alternated between compensation voltages (CV) of −50 and −70, with identical data-dependent settings applied at both CVs. The precursor ion mass spectra were collected at a resolution of 120,000 across an *m*/*z* range of 375–1375. Using a cycle time of 1.5 s, the most abundant precursors with charges from 2 to 7 were isolated with an *m*/*z* window of 0.7 and fragmented by collision-induced dissociation (CID) at 35%. Fragment spectra were recorded in the ion trap at a rapid scan rate. Dynamic exclusion was set to 60 s. The ten most abundant MS2 fragment ions were isolated using multinotch isolation for further MS3 fragmentation. MS3 fragmentation was performed using higher-energy collision dissociation (HCD) at 55%, and the MS3 spectra were recorded in the Orbitrap at a resolution of 50,000 and within an *m*/*z* range 100–500.

Raw files were processed and analyzed using Proteome Discoverer v.3.0 (Thermo Fisher Scientific; MA; USA). The data were matched against the RefSeq protein sequences of CCUG 70745 (PGAP v. 4.2; 4806 sequences) with Sequest HT as the search engine, using a precursor tolerance of 5 ppm and a fragment ion tolerance of 0.6 Da. Tryptic peptides were accepted with one missed cleavage. Methionine oxidation and acetylation on protein N-termini were set as variable modifications, while cysteine carbamidomethylation, TMTpro on lysine, and peptide N-termini were set as fixed modifications. Percolator was used for PSM validation with a strict FDR threshold of 1%. For quantification, TMT reporter ions were identified in the MS3 HCD spectra with a 3 mmu mass tolerance, and the TMT reporter intensity values for each sample were normalized on the basis of the total peptide amount. The SPS threshold was set to 65%, and a Sequest HT threshold score of 2 was chosen. Only unique peptides were used for relative quantification, and proteins were required to pass a protein FDR of 1%.

### Statistical Rationale

The quantified proteins were analyzed in RStudio v. 4.4.1. The normalized abundances of proteins identified and quantified with two or more peptides were log2-transformed, and principal component analysis (PCA) was performed, using the *prcomp* function to cluster conditions and identify potential outliers. Relative abundances and fold-changes were calculated by comparing each condition to the mock control. The requirement of at least two peptides per protein is a conservative criterion that reduces false identifications and yields more robust TMT quantification, providing more reporter-ion observations per protein and lower ratio variance, which supports confident fold-change estimation in a complex bacterial proteome. The threshold for identifying differentially expressed proteins (DEPs) was set at a fold-change of ±1.5. The significance of these proteins was defined by a single criterion applied consistently throughout: a fold-change of ±1.5 together with a Welch́s *t*-test nominal *p*-value < 0.05. The *p*-values were additionally adjusted for multiple testing by calculating the false discovery rate (FDR) using the Benjamini–Hochberg method; however, multiple-testing corrections in quantitative proteomics, while useful, can be overly conservative and mask biologically meaningful abundance changes,^14^ and the FDR is reported alongside each *p*-value for transparency but was not used as an additional filtering threshold for calling differentially expressed proteins. The differentially expressed proteins were visualized with heatmaps and volcano plots generated by the heatmap and EnhancedVolcano functions, respectively. All plots were created using the *ggplot2* program. Venn diagrams were generated with *ggvenn*, showing the number of shared and unique DEPs for each condition. A soft clustering analysis was performed using all quantified proteins with Mfuzz Analysis (https://omia.untangledbio.com/mfuzz/) via the Untangled Biosciences web interface.

### Functional Annotation of the Identified Proteins and Database Search

The functional annotation of the proteins identified in the quantitative proteomics experiment was performed as in Salvà-Serra et al. (2023).^15^ Briefly, OmicsBox v. 3.3.2 (BioBam Bioinformatics S.L., Valencia, Spain) and its plugins for Gene Ontology annotation (Blast2GO v. 2022.0815),^16^ protein family assignment (InterproScan v. 5.70–102.016),^17^ and clustering of orthologous groups (EggNOG-Mapper 2.1.0 with EggNOG v. 5.0.217)^18^ were used. Only the proteins annotated by the EggNOG mapper with experimental evidence and one-to-one orthology were considered. Furthermore, redundancies were removed using the GO True Path Rule and the taxon: Gammaproteobacteria (Taxonomy ID: 1236).^19^ Enzyme Commission (EC) numbers and metabolic pathways from the Kyoto Encyclopedia of Genes and Genomes (KEGG)^20^ were assigned to the filtered results to obtain clusters of orthologous groups (COG) categories.

The differentially expressed proteins identified in this study were further analyzed by using multiple databases for various purposes. For protein–protein interaction analysis, the DEPs at both antibiotic concentrations were searched against the STRING database.^21^ To determine the subcellular localization of the proteins, we used DeepLocPro.^22^ Specific databases for antimicrobial resistance (CARD^23^) and *E. coli* metabolism (EcoCyc^24^) were consulted to identify the possible functions of the DEPs produced under each condition.

### Enrichment Analysis

Gene Set Enrichment Analysis (GSEA) v.4.2.2 was performed using the GO terms and annotated pathways identified from the proteomics experiment with OmicsBox v.3.2.4.^25^ Therefore, the proteins detected under each condition were ranked by fold change and p value to identify sets of genes and gene networks that responded to the different conditions. The default settings and 1000 permutations were maintained for the analysis. Sets of proteins with a *q*-value of < 0.05 were considered significantly enriched. *ggplot2* was used to generate bubble plots of the enriched GO terms and pathways identified in the study, following a previously described protocol.^26^

## Results

### Antibiotic Susceptibility Testing

According to the antibiotic susceptibility test of *E. coli* CCUG 70745, the strain was resistant to 20 of the 25 antibiotics tested in the *Enterobacteriales* panel, including various beta-lactams, an aminoglycoside, fluoroquinolones, and antifolates (Table S1). The strain was susceptible to aminoglycosides amikacin and gentamicin, polymyxin (colistin), tetracycline (tigecycline), and nitrofurantoin. The strain was resistant to the three tested carbapenems. However, the minimal inhibitory concentration for Meropenem was only reported as >16 μg/mL and was not precisely determined. Given the importance of Meropenem in clinical practice, MIC analysis was performed using Meropenem concentrations ranging from 4 μg/mL to 2048 μg/mL, in accordance with EUCAST recommendations. The MIC of Meropenem for *E. coli* strain CCUG 70745 was 128 μg/mL, which is four times higher than that of the carbapenem-resistant strain used as a control (Figure S1A).

Concentrations at and above the MICs (128 μg/mL and 192 μg/mL, respectively) were selected to perturb the resistant phenotype at levels known to be inhibitory for this strain; clinically typical serum concentrations of Meropenem are well below this isolate’s MIC. However, according to our experiment (Figure S1B,C), concentrations below 128 μg/mL produced no measurable effect on growth, whereas concentrations above 192 μg/mL significantly affected the culture, extending recovery time after treatment by 105 min (Figure S1B,C). The brief 15 min exposure was deliberately chosen to capture the immediate, primary proteomic response, while minimizing confounding secondary adaptations and cell death associated with longer exposures.

### Global Proteome Response of *E. coli* CCUG 70745 to Meropenem Is Dose-Dependent

To investigate the global responses to Meropenem and its impact on the metabolism of the multidrug-resistant *E. coli* CCUG 70745, the bacterial culture in the mid log phase was exposed to two different concentrations of Meropenem: 128 μg/mL and 192 μg/mL. The samples were then analyzed using tandem mass spectrometry with TMT labeling for protein quantification. As a result, 35,708 peptides were identified. Additionally, 2951 proteins, representing 61.4% of the strain’s theoretical proteome (4850 proteins) and detected with more than one peptide across all tested conditions, were identified and quantified. Approximately 39 proteins were identified but could not be quantified; these proteins were excluded from the differential expression analysis. The complete list of quantified proteins, their abundances, and functional annotations can be found in Table S2.

A PCA was conducted to examine the impact of different antibiotic concentrations used in the proteomics experiment. The first two principal components accounted for 66% of the variance in the proteomics data, indicating that antibiotic-treated samples and controls were clearly separated. The PCA score plot in Figure 1A shows four distinct clusters that group all biological replicates by condition: control 1, control 2, 128 μg/mL, and 192 μg/mL, with no outliers or mixed data points. The experimental condition that showed the greatest variation among all was the group treated with 192 μg/mL of Meropenem.

**Figure 1:**
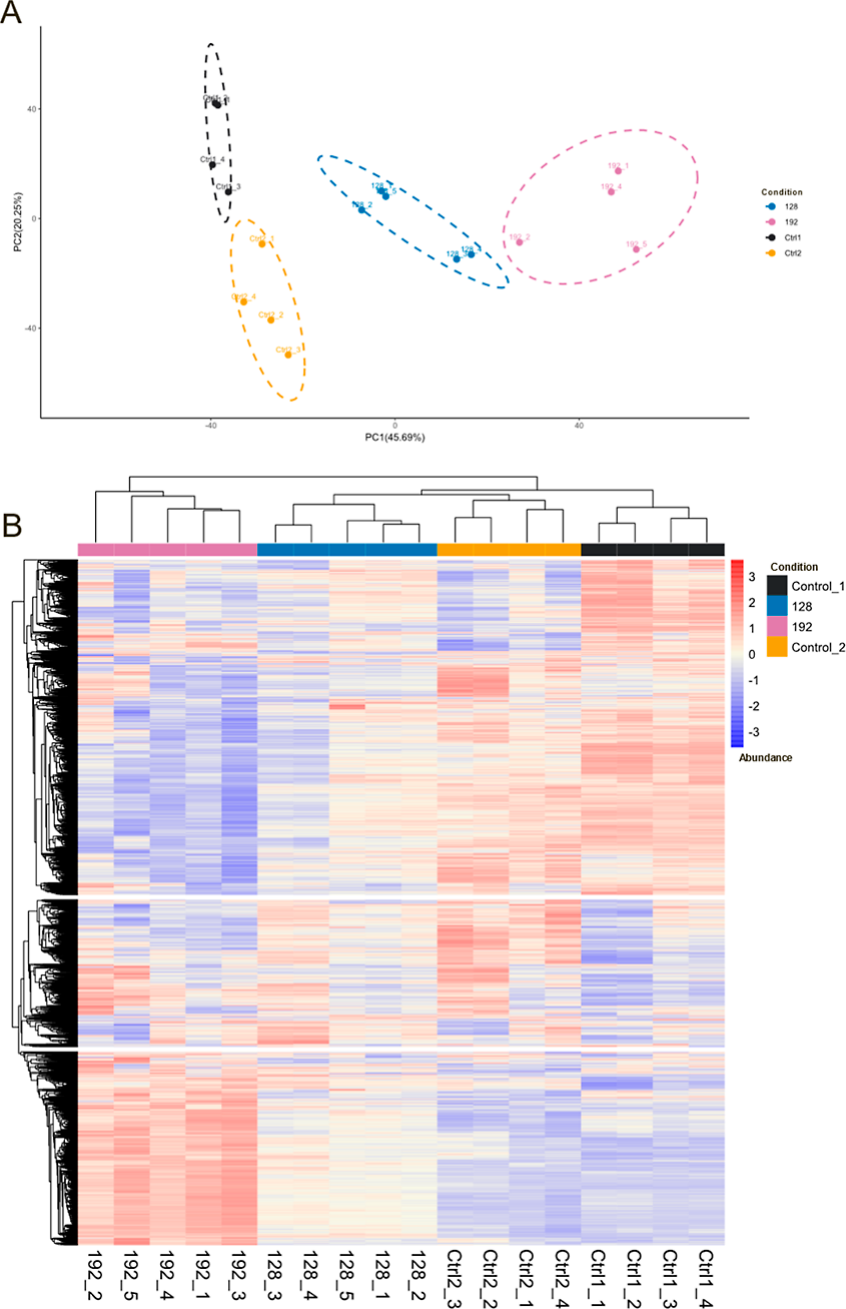
Protein profile, replicate variability, and grouping of the quantified proteins from *E. coli* CCUG 70745 under exposure to Meropenem. (A) PCA scatter plot showing four different clusters in the first two principal components of the analysis. (B) Heatmap and dendrogram of the normalized abundances of the quantified proteins across the four tested conditions, grouped by Euclidean clustering. In red, high abundances are represented, and in blue, low abundances are represented. The black band represents the replicates from control_1; in yellow, the replicates from control_2 are shown; in light blue, the replicates of the treatment with 128 μg/mL of Meropenem are clustered; and under pink, the replicates of the treatment with 192 μg/mL of Meropenem are clustered. The PCA and heatmap together illustrate that samples exposed to 192 μg/mL Meropenem form a distinct cluster characterized by broad remodeling of the proteome, whereas 128 μg/mL produces a more limited shift that remains closer to the mock controls.

In Figure 1B, the clustered heatmap shows two distinct groups based on the expression profiles of the quantified proteins: on the right, the controls cluster with the 128 μg/mL condition, and on the left, the samples treated with 192 μg/mL Meropenem. Interestingly, the samples treated with 128 μg/mL Meropenem exhibit a protein expression profile more similar to that of the mock control. Three main protein sets were identified, consisting of 1,465, 643, and 843 proteins, respectively, whose expression differed between the controls and the samples from the 192 μg/mL group. These global patterns indicate that even a brief 15 min exposure establishes a clear, dose-dependent proteomic signature, with higher Meropenem concentrations driving a broad rewiring of cellular functions.

### Proteins from Cell Envelope and Proteostasis Stress Modules Are Rapidly Activated as Response to Meropenem

Protein abundances were measured relative to the mock control (no antibiotic added; incubated for 15 min). Differentially expressed proteins (DEPs) were identified with a fold change (FC) > 1.5 and a *p*-value < 0.05. In the 128 μg/mL condition, 172 proteins met these criteria, while in the 192 μg/mL condition, 813 proteins were identified as DEPs (Figure 2A and Table S3). As expected, the number of DEPs showed a dose-dependent increase. Generally, the proportion of proteins showing an increase in expression was higher than that of proteins demonstrating lower levels of expression; 101 proteins demonstrated higher levels of expression at 128 μg/mL and 639 proteins at 192 μg/mL, including (Figure 2B up). Among the proteins with increased abundance, several formed a clear envelope- and proteostasis-oriented stress module, including the phage shock protein PspC, the periplasmic chaperone Spy, the small heat-shock protein IbpB, and multiple outer-membrane or periplasm-associated lipoproteins such as YcfJ, YgdI, YgdR, YceB, Blc, and MliC, together with the uncharacterized proteins YgaC, YgaM, and Alx. Many of these proteins showed higher fold-changes at 192 μg/mL than at 128 μg/mL, indicating a dose-dependent reinforcement of cell-envelope integrity and protein quality-control mechanisms during early Meropenem exposure.

**Figure 2:**
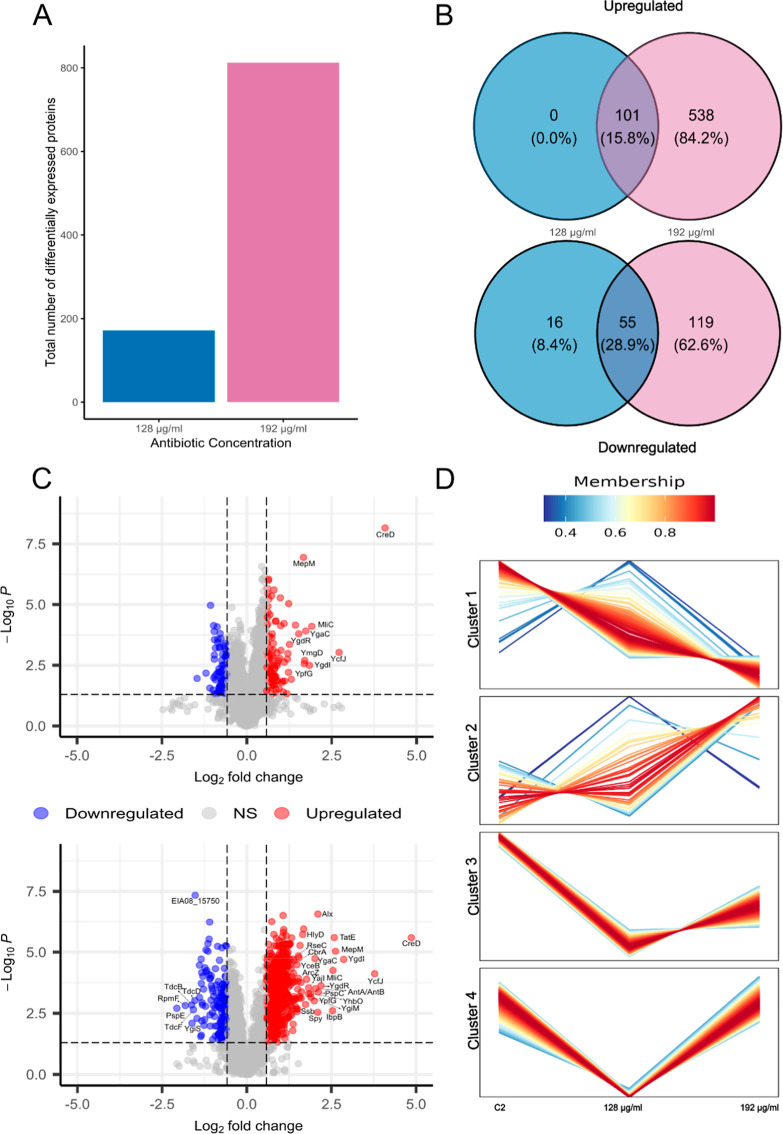
Differentially expressed proteins (DEPs) of *E. coli* CCUG 70745 after exposure to Meropenem at 128 μg/mL and 192 μg/mL. (A) Number of differentially expressed proteins. In light blue: 128 μg/mL; in pink: 192 μg/mL. (B) Common DEPs at 128 μg/mL and 192 μg/mL Meropenem exposure. The Venn diagram on top displays the proteins exhibiting higher levels of expression, and the Venn diagram at the bottom represents the proteins with lower levels of expression. (C) Volcano plots showing the greatest numbers of DEPs. *X*-axis log2 FC, *Y*-axis −log10 *p*-value. Displayed in red are the proteins exhibiting higher levels of expression and in blue are the proteins with lower levels of expression. The upper plot shows DEPs at 128 μg/mL; the bottom plot shows DEPs at 192 μg/mL. (D) Soft clustering of all the proteins identified in the quantitative proteomics analysis. Mfuzz representation of the four clusters identified. Clusters 1–4, respectively, group proteins with decreasing abundance (housekeeping and information processing functions) and increasing abundance (cell envelope biogenesis, efflux and transport, signaling, and stress response proteins), emphasizing a redistribution of resources from growth to envelope-centered defense as Meropenem concentration increases. Differentially expressed proteins were defined by |fold change| ≥1.5 and Welch’s *t*-test *p* < 0.05; TMT reporter-ion intensities were normalized to total peptide amount and log_2_-transformed prior to analysis.

Regarding proteins exhibiting lower levels of expression, 71 displayed lower expression at 128 μg/mL and 174 proteins at 192 μg/mL. Samples treated with both antibiotic concentrations shared 28.9% of the proteins (Figure 2B down). To visualize proteins with the largest fold changes, volcano plots were generated (Figure 2C), highlighting those with the most significant fold changes at both antibiotic concentrations (Table 1). Proteins, such as cell envelope integrity protein CreD, glycine zipper 2TM domain-containing protein YcfJ, YgdI/YgdR family lipoprotein YgdI, murein dd-endopeptidase MepM, C-type lysozyme inhibitor MliC, lipoprotein YgdR, and DUF2002 family protein YgaC, were highly expressed at both antibiotic concentrations, with higher fold-changes observed at the increased antibiotic level. Some stress-response proteins, such as the small heat-shock protein IbpB, followed the same directional trend but reached statistical significance only at the higher dose: IbpB increased modestly at 128 μg/mL (FC = 1.58; *p* = 0.28) and was strongly and significantly induced at 192 μg/mL (FC = 5.79; *p* = 2.5 × 10^–3^), indicating a consistent, dose-dependent upregulation that reaches significance at 192 μg/mL. Regarding the proteins exhibiting lower expression, most showed a negative trend from 128 μg/mL to 192 μg/mL, with even larger fold changes at the highest antibiotic concentration. Beyond the increase in DEP numbers, many core information processing proteins involved in translation, DNA replication, and transcription already showed a downward trend at 128 μg/mL that became more pronounced at 192 μg/mL, suggesting an early shift toward growth arrest and resource reallocation.

**Table 1:** Common DEPs with the Largest Fold-Changes at Two Different Meropenem Concentrations^a^

accession	UniProt	protein name	description	128 μg/mL				192 μg/mL				subcellular localization
				fold change	*p*-value	FDR	expression	fold change	*p*-value	FDR	expression	
**WP_000920306.1**	P08369	**CreD**	**cell envelope integrity protein**	16.81	7.09 × 10^–9^	2.09 × 10^–5^	higher	29.02	2.54 × 10^–6^	4.48 × 10^–4^	higher	cytoplasmic membrane
**WP_001043458.1**	P0AB35	**YcfJ**	**glycine zipper 2TM domain-containing protein**	6.58	9.32 × 10^–4^	1.11 × 10^–2^	higher	13.71	7.54 × 10^–5^	1.08 × 10^–3^	higher	outer membrane
**WP_000750398.1**	P65292	**YgdI**	**uncharacterized lipoprotein Ygdl**	3.58	3.20 × 10^–3^	2.39 × 10^–2^	higher	7.27	1.97 × 10^–5^	6.59 × 10^–4^	higher	outer membrane
**WP_001184045.1**	P0AFS9	**MepM**	**Murein** **dd** **-endopeptidase MepM**	3.17	1.15 × 10^–7^	1.70 × 10^–4^	higher	6.16	9.11 × 10^–6^	5.54 × 10^–4^	higher	cytoplasmic membrane
WP_000503931.1	P0A843	TatE	sec-independent protein translocase protein TatE	2.69	7.04 × 10^–5^	3.06 × 10^–3^	higher	6.01	2.51 × 10^–6^	4.48 × 10^–4^	higher	cytoplasmic membrane
**WP_000061974.1**	**A0A066SZP2**	**MliC**	**lysozyme inhibitor. Putative lipoprotein**	3.76	7.92 × 10^–5^	3.18 × 10^–3^	higher	5.85	5.51 × 10^–5^	9.46 × 10^–4^	higher	outer membrane
WP_001243431.1	P0C058	IbpB	small heat shock protein IbpB	1.58	2.82 × 10^–1^	4.05 × 10^–1^	higher	5.79	2.48 × 10^–3^	7.92 × 10^–3^	higher (trend; n.s.)	cytoplasmic
**WP_000758655.1**	P65294	**YgdR**	**uncharacterized lipoprotein YgdR**	2.88	1.61 × 10^–4^	4.17 × 10^–3^	higher	4.56	2.35 × 10^–4^	1.78 × 10^–3^	higher	outer membrane
WP_000907387.1	P0AFN2	PspC	phage shock protein C	1.49	1.80 × 10^–2^	6.77 × 10^–2^	higher	4.32	4.30 × 10^–4^	2.54 × 10^–3^	higher	cytoplasmic membrane
WP_001098826.1	Q8FDE1	Alx	putative membrane-bound redox modulator Alx	2.13	6.01 × 10^–5^	2.86 × 10^–3^	higher	4.28	2.71 × 10^–7^	3.05 × 10^–4^	higher	cytoplasmic membrane
WP_001228990.1	A0A0A1A8M7	Spy	periplasmic chaperon Spy	2.48	1.20 × 10^–2^	5.37 × 10^–2^	higher	4.26	2.89 × 10^–3^	8.87 × 10^–3^	higher	periplasmic
WP_033554326.1	WP_033554326.1	-	AntA/AntB antirepressor family protein	2.34	6.20 × 10^–3^	3.58 × 10^–2^	higher	4.08	3.06 × 10^–4^	2.07 × 10^–3^	higher	cytoplasmic
**WP_000281320.1**	P0AD53	**YgaC**	**uncharacterized protein YgaC**	3.33	1.23 × 10^–4^	3.73 × 10^–3^	higher	4.04	1.81 × 10^–5^	6.59 × 10^–4^	higher	cytoplasmic
**WP_001270513.1**	**A0A0A1AAR6**	**YpfG**	**DUF1176 domain containing protein**	3.23	2.73 × 10^–3^	2.16 × 10^–2^	higher	3.98	9.82 × 10^–4^	4.32 × 10^–3^	higher	periplasmic
WP_021558026.1	A0AAX4M0E3	-	helicase subunit of the DNA excision repair complex	2.08	1.41 × 10^–2^	5.84 × 10^–2^	higher	3.71	7.04 × 10^–4^	3.54 × 10^–3^	higher	cytoplasmic
WP_000037592.1	A0A0A1A9S8	YhbO	glutamine amidotransferase	2.15	2.38 × 10^–3^	2.03 × 10^–2^	higher	3.69	4.99 × 10^–4^	2.79 × 10^–3^	higher	cytoplasmic
WP_001298536.1	A0A067HJP2	D3C88_20350	DUF3251 domain containing protein	2.31	1.03 × 10^–3^	1.17 × 10^–2^	higher	3.54	2.82 × 10^–4^	1.98 × 10^–3^	higher	outer membrane
WP_001125331.1	P0ADT8	YgiM	uncharacterized protein YgiM	2.29	1.78 × 10^–3^	1.68 × 10^–2^	higher	3.54	5.11 × 10^–4^	2.85 × 10^–3^	higher	cytoplasmic membrane
WP_000891515.1	P0AAW9	AcrZ	multidrug efflux pump accessory protein AcrZ	2.39	4.39 × 10^–4^	7.16 × 10^–3^	higher	3.40	1.27 × 10^–4^	1.34 × 10^–3^	higher	cytoplasmic membrane
WP_110221346.1	WP_110221346.1	-	single-stranded DNA-binding protein	2.17	1.41 × 10^–2^	5.84 × 10^–2^	higher	3.33	1.38 × 10^–3^	5.29 × 10^–3^	higher	cytoplasmic
WP_000070117.1	A0A0A1A1U0	EIA08_01220	probable multidrug ABC transporters permease YbhS	2.00	7.68 × 10^–4^	9.72 × 10^–3^	higher	3.20	1.12 × 10^–6^	4.46 × 10^–4^	higher	cytoplasmic membrane
WP_001361294.1	A0A0A1A2M8	YbhG	UPF194 membrane protein YbhG	1.96	1.62 × 10^–3^	1.58 × 10^–2^	higher	3.14	1.88 × 10^–6^	4.48 × 10^–4^	higher	cytoplasmic membrane
WP_000527844.1	P0ABU7	ExbB	biopolymer transport protein ExbB	1.86	2.61 × 10^–3^	2.12 × 10^–2^	higher	3.06	4.14 × 10^–4^	2.50 × 10^–3^	higher	cytoplasmic membrane
WP_001295174.1	P0ADQ7	YgaM	uncharacterized protein YgaM	1.82	3.12 × 10^–2^	9.58 × 10^–2^	higher	3.05	7.72 × 10^–4^	3.73 × 10^–3^	higher	cytoplasmic membrane
WP_000019197.1	P0AA47	PlaP	low-affinity putrescine importer PlaP	1.48	2.53 × 10^–2^	8.40 × 10^–2^	-	3.03	1.73 × 10^–4^	1.51 × 10^–3^	higher	cytoplasmic membrane
WP_000780581.1	A0A0A1A117	Blc	outer membrane lipoprotein Blc	1.99	7.44 × 10^–5^	3.06 × 10^–3^	higher	2.98	5.27 × 10^–6^	4.48 × 10^–4^	higher	outer membrane
WP_110221298.1	WP_110221298.1	YfdP	protein YFdP	1.34	7.95 × 10^–2^	1.68 × 10^–1^	-	2.95	6.37 × 10^–4^	3.26 × 10^–3^	higher	cytoplasmic
WP_000973081.1	A0A066RN61	FadE	acyl-coenzyme A dehydrogenase	1.69	2.14 × 10^–2^	7.52 × 10^–2^	higher	2.94	7.63 × 10^–4^	3.70 × 10^–3^	higher	cytoplasmic membrane
WP_022646303.1	A0A0A1ADU8	CbrA	protein CbrA	2.35	9.22 × 10^–6^	1.09 × 10^–3^	higher	2.91	1.68 × 10^–5^	6.59 × 10^–4^	higher	cytoplasmic membrane
WP_022645995.1	A0A0A1A8J5	RseC	SoxR-reducing system protein RseC	1.98	5.30 × 10^–6^	9.19 × 10^–4^	higher	2.88	1.43 × 10^–5^	6.59 × 10^–4^	higher	cytoplasmic membrane
WP_001295443.1	P0AB26	YceB	uncharacterized lipoprotein YceB	1.85	9.68 × 10^–4^	1.13 × 10^–2^	higher	2.88	1.15 × 10^–4^	1.26 × 10^–3^	higher	outer membrane
WP_000471889.1	P0ABB8	MgtA	magnesium-transporting ATPase	1.84	1.40 × 10^–2^	5.84 × 10^–2^	higher	2.85	1.58 × 10^–4^	1.47 × 10^–3^	higher	cytoplasmic membrane
WP_000620399.1	E2QEP0	YrbL	protein	1.31	2.68 × 10^–3^	2.14 × 10^–2^	-	2.84	3.25 × 10^–4^	2.12 × 10^–3^	higher	cytoplasmic membrane

a Proteins are listed when they meet the criterion of fold-change ±1.5 and Welch́s *t*-test *p* < 0.05; the false discovery rate (FDR, Benjamini-Hochberg) is shown for reference but was not used as a filtering threshold (see Methods). “Higher”/“Lower” denotes the direction of change in each condition; entries marked as a trend (n.s.) at 128 μg/mL did not reach *p* < 0.05 at that concentration but showed the same direction of change and were significant at 192 μg/mL. Highlighted in bold are the proteins demonstrating higher expression in both 128 μg/mL and 192 μg/mL.

A soft clustering analysis with Mfuzz was conducted on the complete protein set to evaluate how proteins with similar expression patterns, considered to be coexpressed, behaved at the two tested antibiotic concentrations (Figure 2D). Four groups were identified, each consisting of 201, 50, 218, and 596 proteins (Table S4). The first cluster comprises proteins that exhibited a decreasing expression trend from 128 μg/mL to 192 μg/mL. This group includes ribosomal proteins, proteins involved in DNA replication and translation, some regulatory proteins, and two-component system proteins. In cluster number two, proteins exhibited an increasing expression pattern, including those involved in cell envelope integrity—such as cell wall, periplasm, and membrane—as well as stress response proteins, ions, sugars, small-molecule transporters and efflux pumps that also exhibited small-molecule transporters and efflux pumps rising pattern. The third and fourth clusters of proteins exhibited slight increases in expression from 128 μg/mL to 192 μg/mL. These clusters included proteins with functions similar to those in the second cluster, such as proteins involved in the divisome, RNA degradation, and the biosynthesis of valine, leucine, and isoleucine. A notable feature of group four is the presence of ABC transporters, two-component system proteins, oxidative phosphorylation proteins, and lipopolysaccharide proteins, which show increased expression at higher concentrations of Meropenem.

Cluster 1 was enriched for ribosomal proteins, replication, and transcription factors with decreasing abundance at higher antibiotic concentrations, whereas clusters 2–4 contained membrane and periplasmic proteins, efflux pumps, transporters, and cell envelope enzymes whose expressions increased with dose, indicating a coordinated trade-off between growth and envelope-centered stress adaptation.

Notably, many enzymes involved in peptidoglycan biosynthesis and remodeling (for example, MurJ, MrdA, AmiD, and DacA), lipid A and LPS core modification (RfaQ/Y, EptA/CptA, and Kdo transferases), and Lpt, Mla, and Tol Pal envelope maintenance systems were concentrated in the upregulated clusters at 192 μg/mL, together supporting an extensive reinforcement and restructuring of the outer cell envelope under high Meropenem pressure.

Together with changes in cell-division and cell-wall-associated proteins, these increases suggest that Meropenem rapidly activates a coordinated envelope-stress and protein quality-control program that stabilizes the cell surface and mitigates misfolded-protein toxicity during early carbapenem exposure.

### Upregulation of Proteins Associated with Transport, Efflux, and Ion Homeostasis Systems Promotes the Survival of the *E. coli* Strain in the Presence of Meropenem

The differentially expressed proteins and clustering analysis also revealed strong activation of transport and efflux systems, including multiple multidrug efflux pumps, outer-membrane channels, and ABC transporters for small molecules and metals, alongside an array of cation and mechanosensitive transporters that increased in abundance with the Meropenem dose.

DEPs at both antibiotic concentrations were searched in the CARD database^23^ using RGI to identify the proteins involved in classical antibiotic resistance mechanisms. For the 128 μg/mL condition, six proteins were identified: three were efflux pumps, exhibiting higher expression, and three were beta-lactamases, with lower expression. At the Meropenem concentration of 192 μg/mL, 23 proteins were identified in CARD; the beta-lactamases CTX-M-15, OXA-1, and BlaEC, as well as CMY-6, were expressed to a lesser degree, while the multidrug transporters were more abundant. The proteins with higher expression levels primarily belonged to two resistance mechanisms: antibiotic efflux, which accounted for most of them, and antibiotic target alteration. Antibiotic efflux was the most common mechanism of resistance in *E. coli* 70745. The outer membrane channel protein TolC demonstrated significantly higher expression at the highest antibiotic concentration, along with AcrAB, AcrEDF, and EmrA from the EmrAB operon, YojI, and MdfABC. Proteins from the two-component systems pathway that control metabolic rewiring in *E. coli* related to antimicrobial resistance, such as ArcAB and CpxA, were highly expressed, as shown in Table S5. These data indicate that the resistant strain counters Meropenem stress not only by upregulating multidrug efflux but also by actively managing membrane potential, osmotic balance, and metal homeostasis, thereby supporting survival under perturbed envelope and energy conditions.

### Meropenem Induced an Overrepresentation in Cell Wall/Membrane/Envelope Biogenesis, Inorganic Ion Transport, Signal Transduction, and Intracellular Trafficking, Secretion, and Vesicular Transport in *Escherichia coli*

Of the 2951 quantified proteins, 2918 were assigned to a cluster of orthologous groups (COG) category using the eggNOG database and COG classifier and served as the background data set for calculating the overrepresentation of DEPs from 128 μg/mL and 192 μg/mL. The 39 proteins not associated with any COG category were hypothetical proteins. The largest COG categories at 128 μg/mL were function unknown (S; 38), nucleotide transport and metabolism (F; 25), carbohydrate transport and metabolism (G; 22), and inorganic ion transport and metabolism (P; 17). At 192 μg/mL, the main COG categories included function unknown (S; 117), cell wall/membrane/envelope biogenesis (M; 108), inorganic ion transport and metabolism (P; 98), and energy production and conversion (C; 58), which accounted for most of the DEPs.

Among the COG terms grouping DEPs, under 128 μg/mL Meropenem stress, proteins from 17 COG categories exhibited higher expression, whereas only 12 COG categories demonstrated lower expression. According to the Fisher exact test performed to identify the overrepresented terms among the DEPs (Table S6), categories such as intracellular trafficking, secretion, and vesicular transport (*U*), defense mechanisms (*V*), and cell wall/membrane/envelope biogenesis (*M*) were significantly overrepresented in the set of proteins showing higher abundance, while in the set of proteins showing lower abundance, nucleotide transport and metabolism (*F*) was significantly overrepresented (Figure 3A). At 192 μg/mL, the DEPs included 19 terms with higher expression and 18 with lower abundance. Four COG categories were significantly overrepresented in the upregulated DEPs: cell wall/membrane/envelope biogenesis (*M*); inorganic ion transport and metabolism (*P*); intracellular trafficking, secretion, and vesicular transport (*U*); and signal transduction mechanisms (*T*). The COG category defense mechanisms (*V*) were also overrepresented. Among the DEPs showing lower abundance, carbohydrate transport and metabolism (*G*) was significantly overrepresented (Figure 3B).

**Figure 3:**
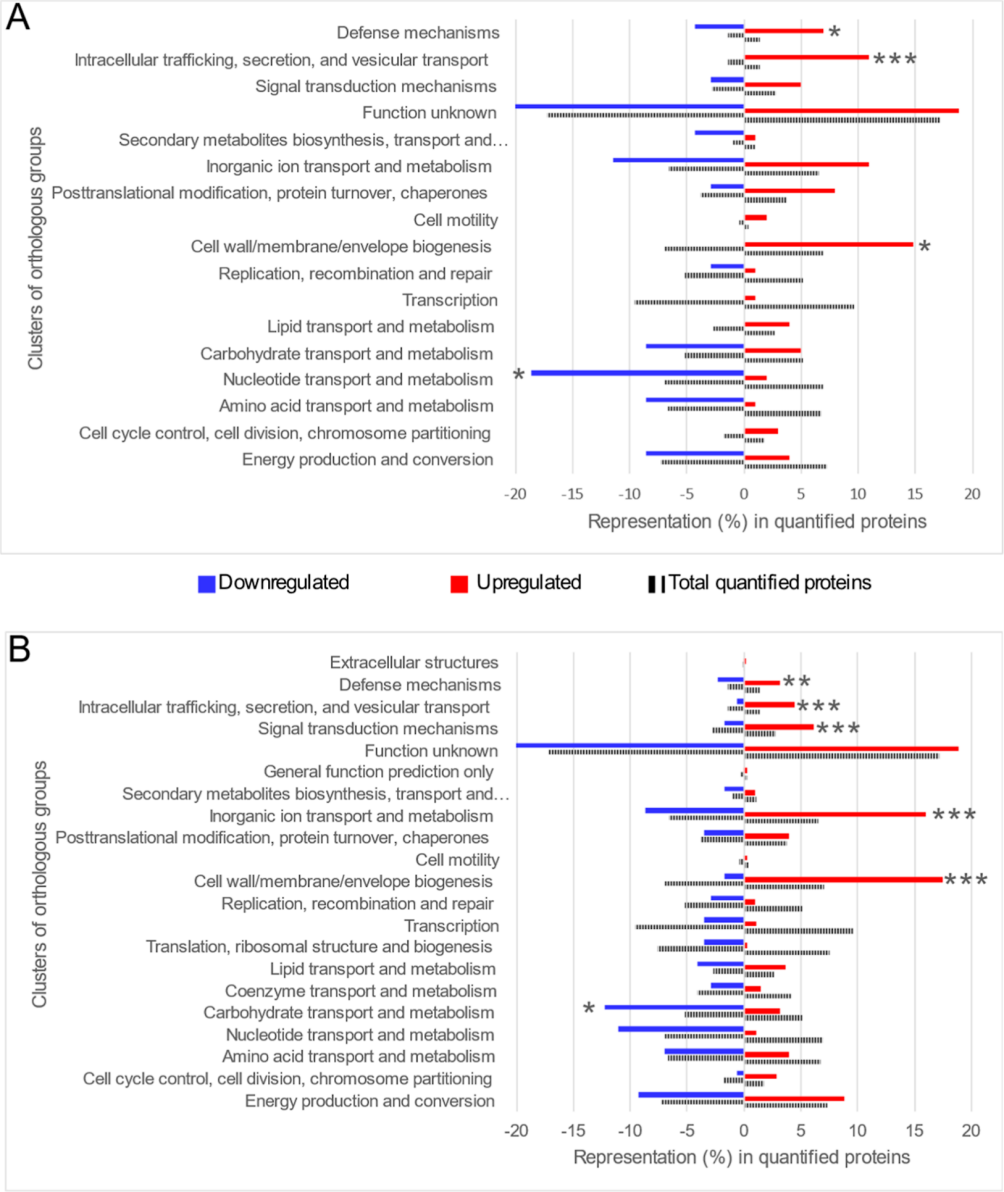
Overrepresented COG categories of the differentially expressed proteins (DEPs) under exposure to Meropenem. (A) 128 μg/mL. (B) 192 μg/mL. Displayed in red are the overrepresented DEPs demonstrating higher expression; in blue, the overrepresented DEPs exhibiting lower expression. The striped bars represent the COG classification of all the quantified proteins in the proteomics experiment.

### Two-Component and c-di-GMP Signaling Networks Show Coordinated Abundance Changes

Consistent with the enrichment of two-component system pathways, several sensor-response regulator pairs involved in envelope, metabolism, and metal-ion stress, together with multiple GGDEF- and EAL-domain proteins that modulate c-di-GMP, showed altered abundance in a Meropenem-dose-dependent manner.

A network analysis of differentially expressed proteins in the STRING database was conducted to examine associations and relationships among proteins (Table S7). The analysis included functional, physical, and high-confidence interactions. As observed in Figure 4A at the 128 μg/mL condition, the proteins showing higher expression are associated with the bacterial membrane and are involved in growth and cell division, polysaccharide biofilm formation, repair and resistance, transport—specifically ABC importers—and oxidative phosphorylation. The proteins showing lower expression fall into five main groups: nucleobase-containing small-molecule metabolic processes; vitamin biosynthetic processes; ABC transporters; sulfur metabolism; and nitrotoluene and formate oxidation.

**Figure 4:**
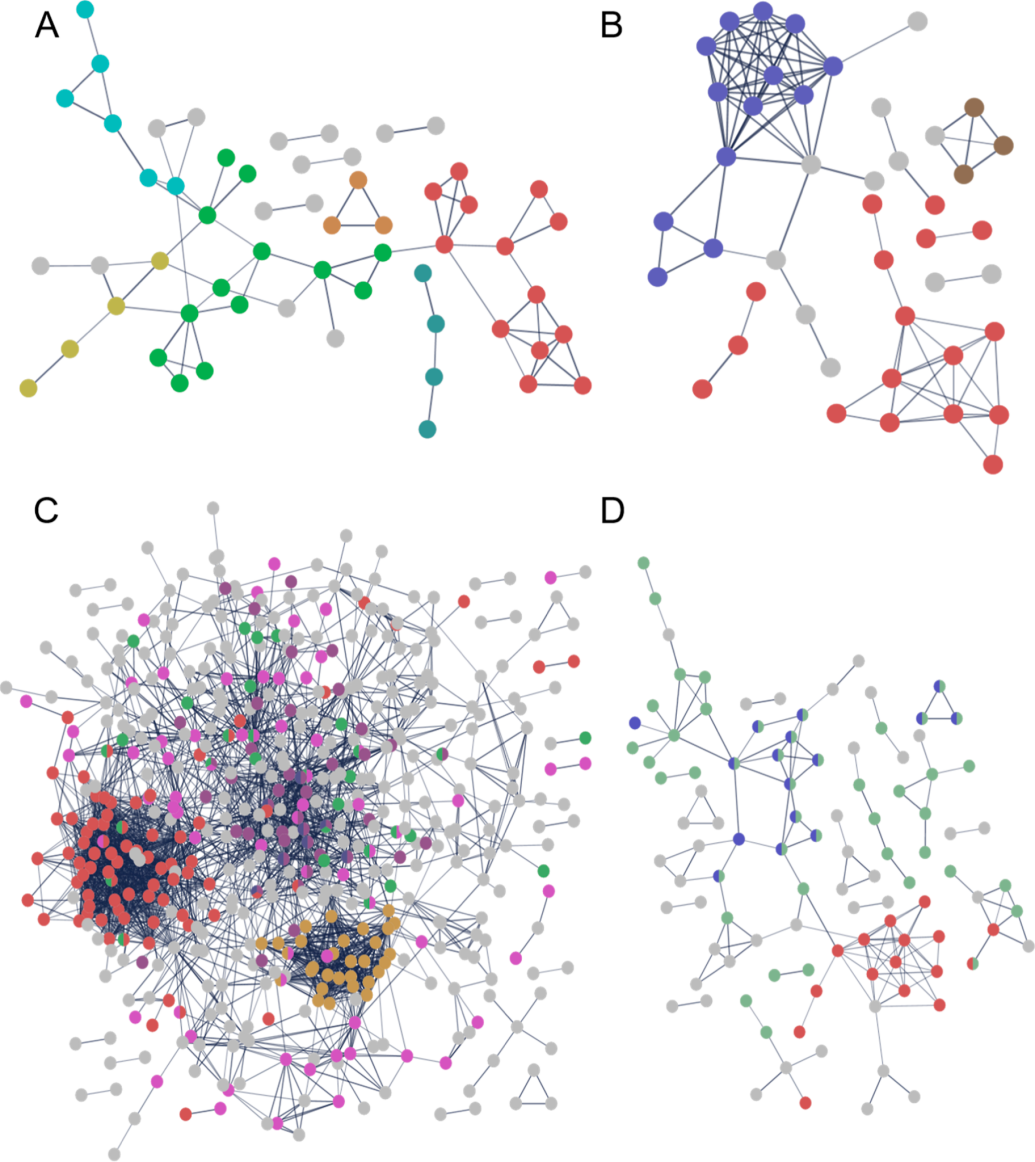
Protein–Protein interaction network based on the differentially expressed proteins at 128 μg/mL and 192 μg/mL performed in the STRING database. (A) 128 μg/mL proteins of higher expression. Red ABC transporters, green resistance and reparation, cyan cell division and growth, yellow biofilm formation, orange energy production, and teal carbohydrate metabolism. (B) 128 μg/mL proteins of lower expression. Red ABC transporters, blue purine metabolism, brown nitrotoluene degradation, and formate oxidation. (C) 192 μg/mL proteins showing higher expression. Red ABC transporters, orange energy production, green resistance, purple cell division and peptidoglycan, navy divisome complex, and fuchsia two-component system. (D) 192 μg/mL proteins showing lower expression. Red ABC transporters and sulfur metabolism, blue nucleobase containing small-molecule metabolic process and vitamin biosynthetic process, and small-molecule metabolic process.

Similarly, at 192 μg/mL, most of the higher-expressing proteins shown in Figure 4C were membrane proteins; among these, three main modules were identified in the protein–protein interaction network. Proteins related to ABC transporters, oxidative phosphorylation, and cell division and shape clustered into distinct groups. In contrast, proteins involved in the two-component system and signaling, as well as those part of the cation antimicrobial peptide resistance pathway, were distributed throughout the network plot. Consistent with this, multiple envelope and metal sensing two-component systems (including CreC, PhoR, CusS, PmrB, and KdpD), together with several diguanylate cyclases and phosphodiesterases (DgcN, DgcQ, DgcZ, PdeB, and PdeF), were significantly induced, consistent with rapid modulation of envelope stress, ion homeostasis, and c-di-GMP signaling networks in response to Meropenem. The signaling changes coincided with strong upregulation of multidrug efflux systems (AcrABEF, MdtA, EmrA/K, MacB, DinF, and TolC) and numerous K^+^, Mg^2+^, Zn^2+^, and proton/cation transporters and mechanosensitive channels (KefB/C, Kdp system, MscS/MscK, CorA, ZntA/ZntB, and Na^+^/H^+^ antiporters), consistent with a role for membrane potential and osmotic control in the adaptive response. The proteins exhibiting lower levels of expression (Figure 4D) were predominantly periplasmic. The clusters of proteins involved in ABC transporters, sulfur metabolism, nucleobase-containing small-molecule metabolic processes, and vitamin biosynthesis are part of the small-molecule metabolic process.

The combined modulation of the two-component and c-di-GMP-processing proteins suggests that Meropenem exposure is sensed as a complex envelope, metal, and osmotic stress that feeds into global regulatory circuits controlling surface properties, biofilm-related behaviors, and gene-expression programs in this carbapenem-resistant strain.

### Cell Outer Membrane, Transmembrane Transport, and ABC-Transporter Activity Are Enriched GO Terms after Meropenem Exposure

The enrichment analysis of GO terms was conducted to identify the cellular components, biological processes, and molecular functions influenced by different concentrations of Meropenem, as shown in Table S8. At 128 μg/mL, 127 specific GO terms were affected; most GO terms showed an increase in expression (positive Normalized Enrichment Score; NES; 86 GO terms). Among these, the cell outer membrane (CC; GO:0009279), xenobiotic transmembrane transporter activity (BP; GO:0042910), and ABC-type transporter activity (MF; GO:0140359) were among the most significant. For the 192 μg/mL condition, 147 GO terms were significant. Amide biosynthetic process (BP; GO:0043604) and aminoacyl-tRNA ligase activity (MF; GO:0004812) were showing lower abundances, while cell outer membrane (CC; GO:0009279), phosphorelay sensor kinase activity (MF; GO:0000155), and xenobiotic transmembrane transport (BP; GO:0006855) were showing higher abundances. Overall, fewer terms were expressed to a higher degree (80) as compared with the 128 μg/mL condition, and the number of terms linked to lower abundances increased (67).

### Enrichment Analysis Highlights the Impact of Meropenem on the Bacterial Secretion System and Oxidative Phosphorylation Pathways

Gene set enrichment and network analyses highlighted extensive changes in respiratory complexes and central metabolism, including components of the electron transport chain as well as enzymes involved in fatty-acid β-oxidation, alternative carbon-source utilization, and cofactor metabolism.

To identify the metabolic pathways affected by Meropenem, the quantified protein set was annotated in the KEGG database, and GSEA analysis was performed (Table S9). In Figure 5, a bubble plot showing the significant pathways for each of the conditions, 128 μg/mL and 192 μg/mL, is presented. The pathway enrichment analysis for the 128 μg/mL condition revealed 55 affected pathways, of which 29 were significantly enriched (Figure 5A). Nine pathways were expressed to a higher degree, and 20 were expressed to a lesser degree. The top five pathways showing significantly higher expression levels included Bacterial secretion system KO03070, Ribosome KO03010, Oxidative phosphorylation KO00190, Cationic antimicrobial peptide resistance KO01503, and Peptidoglycan synthesis KO00550. In line with the enrichment of oxidative phosphorylation, several NADH quinone oxidoreductase subunits, cytochrome d and c oxidases, quinoprotein dehydrogenases, and nitrite/nitrate and DMSO reducing complexes were upregulated together with fatty acid catabolic enzymes (FadA/B/E/I and FadL/D) and trehalose and glycerol 3 phosphate utilization systems, suggesting a shift toward alternative respiratory routes and enhanced lipid utilization under Meropenem-induced cell wall stress. Cysteine and methionine metabolism KO00270, biosynthesis of cofactors KO01240, folate biosynthesis KO00790, drug metabolism—other enzymes KO00983—and nucleotide metabolism KO01232 were the pathways that were most significantly present to a lesser degree. The pathways with the largest number of proteins included thiamine metabolism (KO00730; 250; higher expression), biosynthesis of cofactors (KO01240; 176; lower expression), ABC transporters (KO02010; 131; higher expression) glycine, serine, and threonine metabolism (KO00260; 121; lower expression). The pathway module with the most differentiated pathways was metabolism of cofactors and vitamins, which included nine different pathways, such as thiamine metabolism (KO00730; 250 proteins; higher expression) and biosynthesis of cofactors (KO01240; 176; lower expression). Membrane transport via ABC transporters (KO02010; 131; higher expression) and amino acid metabolism, including glycine, serine, and threonine metabolism (KO00260; 121; lower expression), were among the top pathways in the module list for this condition.

**Figure 5:**
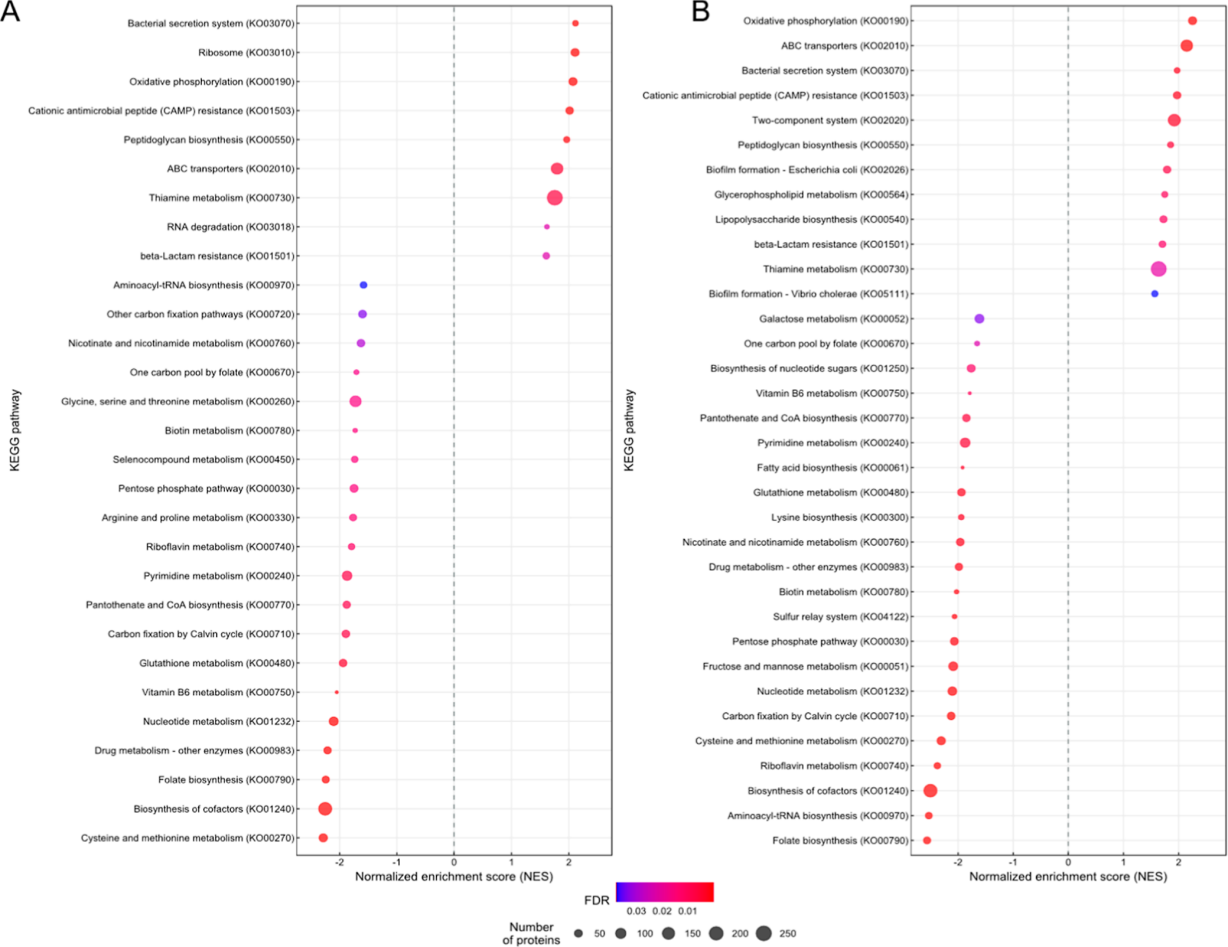
Significantly enriched pathways based on KEGG annotation of the two Meropenem concentrations tested. (A) KEGG enriched pathways at 128 μg/mL. (B) KEGG enriched pathways at 192 μg/mL. The bubble size corresponds to the number of proteins included in the pathway. The color of the bubbles corresponds to the *q*-value obtained for the pathway in the GSEA analysis. The underlying differentially expressed proteins were defined by |fold change| ≥1.5 and Welch’s *t*-test *p* < 0.05; TMT reporter-ion intensities were normalized to total peptide amount and log_2_-transformed prior to analysis.

For the 192 μg/mL condition, 53 pathways were affected, and 34 were significantly enriched (Figure 5B). Pathways, such as Oxidative phosphorylation KO00190, ABC transporters KO02010, Bacterial secretion system KO03070, Cationic antimicrobial peptide (CAMP) resistance KO01503, and Two-component system KO02020, were the top five of the 12 pathways that demonstrated higher levels of expression with NES ≥ 1,9 and the smallest *q*-values. Among the 22 pathways with lower levels of expression, Biosynthesis of cofactors KO01240, Aminoacyl tRNA biosynthesis KO00970, and cysteine and methionine metabolism KO00270 were the least expressed. The pathways with the greatest number of proteins that were upregulated were thiamine metabolism (KO00730; 250), two-component system (KO02020; 146), and ABC transporters (KO02010; 131). Regarding the proteins showing lower expression, the largest number of proteins clustered in the biosynthesis of cofactors (KO01240; 176 proteins), pyrimidine metabolism (KO00240; 85 proteins), and Fructose and mannose metabolism (KO00051; 72 proteins).

Twenty-one of all the significant KEGG pathways were shared between both antibiotic treatments; however, four pathways demonstrated lower expression in 128 μg/mL (arginine and proline metabolism KO00330; selenocompound metabolism KO00450; glycine, serine, and threonine metabolism KO00260; and other carbon fixation pathways (KO00720)), and two pathways were increased (RNA degradation KO03018 and ribosome KO03010). Regarding the 192 μg/mL condition, 13 pathways were unique, with seven showing increased activity and six showing reduction. Among the increased pathways, the two-component system had the most components and was listed first. Meanwhile, carbohydrate metabolism pathways—such as fructose and mannose metabolism and galactose metabolism—were the most prevalent at the bottom of the list.

These findings point to a reorganization of energy metabolism toward alternative respiratory routes and enhanced lipid and carbohydrate utilization, enabling ATP production and redox balance to be maintained while growth-associated biosynthetic processes, including protein synthesis and DNA replication, are downregulated under carbapenem stress.

## Discussion

This study presents the proteomic responses of multidrug-resistant *E. coli* CCUG 70745 to two Meropenem concentrations (128 μg/mL and 192 μg/mL). Quantitative proteomics is a powerful technique that captures the proteome’s expression under specific conditions, providing extensive data that reflects the microorganism’s complex response. This study reports a coverage of 61.4% of the theoretical proteome of the strain studied, demonstrating the usefulness of tandem mass spectrometry combined with tandem mass tags for exploring the mechanisms of antimicrobial resistance. The approach enables detection of proteins conferring a resistant phenotype, including traditional resistance pathways and secondary mechanisms that could increase antimicrobial resistance. The results presented are in line with other quantitative proteomics studies, for example, Liu et al. (2020), which described covering 92% of the proteome of an *E. coli* strain,^27^ while another study, where label-free quantification approaches were employed, allowed the detection of 46% of the *E. coli* proteome in a data-dependent acquisition and tandem mass spectrometry experiment.^28^

The quantitative proteomic data sets from this study provide valuable insights into *E. coli*’s responses to Meropenem. However, due to the high plasticity of the *E. coli* genome, different multidrug resistance phenotypes are expressed;^29^ the results presented are limited to the strain studied and cannot be generalized to other *E. coli* strains. While this study provides 61.4% proteome coverage, a significant number of low-abundance proteins may be undetected. Additionally, post-translational modifications that could affect antibiotic resistance were not considered in the study design. The short 15 min exposure time captures the immediate, primary response but could miss longer-term adaptations, such as biofilm formation or persister states. The present study was designed as a discovery-stage, system-wide characterization of the acute proteome response and the abundance changes reported here describe associations rather than functionally validated mechanisms. Targeted orthogonal validation by, for example, RT-qPCR of marker transcripts, targeted proteomics (parallel- or selected-reaction monitoring) of efflux and envelope proteins, or phenotypic efflux assays and targeted gene knockouts, represents the necessary next step to confirm the functional relevance of these observations. Moreover, as these findings are based on a single carbapenem-resistant *E. coli* isolate (CCUG 70745), generalization to other carbapenem-resistant *E. coli* (CREC) strains should be made cautiously, as resistance mechanisms and proteome responses are known to vary considerably between strains. In addition, individual proteins quantified with relatively high false discovery rate (FDR) values should be interpreted with caution; the most robust conclusions of this study therefore arise from pathway- and systems-level trends rather than from changes in single proteins. However, this study will add to the growing understanding of the complexity of antibiotic resistance mechanisms.

The fluctuations in the fold changes of DEPs at the highest antibiotic concentration were evident for both upregulated and downregulated proteins, as noted in previous research.^11^ The proteins exhibiting the largest fold-changes (33 proteins) were mainly located in the cytoplasmic membrane, outer membrane, and periplasm, likely due to Meropenem’s mechanism of action, which disrupts the cell wall and proteins from the cell membrane in *E. coli*.^30^ Although the strain in the study was multidrug-resistant, the cell envelope was affected. Therefore, proteins such as CreD, an inner membrane protein with a key role in regulating cell envelope integrity and cell division, were highly upregulated. A similar pattern was observed for YcfJ, a structural component of the membrane; YgdI, a cell membrane protein; MepM, an endopeptidase that facilitates cell wall expansion; and TatE, which transports folded proteins across the membrane. As supported by the soft clustering analysis, proteins involved in cell envelope integrity, divisome, stress response, and two-component systems were upregulated in response to Meropenem stress. The two-component system regulates the cell physiology in response to environmental signals,^31^ with one of the components acting as a sensor kinase and the other as a response regulator. Two-component system proteins act together to ensure bacterial adaptation.

*E. coli*’s resistance mechanisms to carbapenems (Meropenem) include the production of carbapenemases or porin alterations that entail structural modifications, which help the bacterium survive longer by repairing itself and conserving energy.^10^ When examining classical resistance mechanisms, strain CCUG 70745 showed a significant increase in antibiotic efflux at the highest Meropenem concentration. Unlike a study by Foudraine et al. (2022), who report the loss of porins OmpC/OmpF and an increase in beta-lactamase production leading to higher resistance,^32^ CCUG 70745 decreased beta-lactamase production and increased the expression of the full machinery needed for assembling AcrAB, AcrDEF, YojI, and TolC complexes. Unlike canonical porin loss in other CREC strains, the dominance of higher-efflux-mechanism expression in CCUG 70745 likely stems from its encoded RND systems (e.g., AcrAB-TolC), which confer additive resistance through efflux activity, estimated to reduce intracellular antibiotic levels. The concurrent decrease in β-lactamase abundance, despite the resistant phenotype, may reflect several nonexclusive factors: (i) an energetic and resource reallocation under acute high-dose stress, in which growth-associated biosynthesis is down-shifted (consistent with the broad reduction of ribosomal, replication, and cofactor-biosynthesis proteins observed here) while efflux and envelope reinforcement are prioritized; (ii) mechanism redundancy, whereby the multiple RND efflux systems carried by this isolate provide additive protection that reduces the marginal benefit of further β-lactamase production at already-saturating hydrolytic capacity; and (iii) regulatory and temporal effects, since the 15 min window captures an early redistribution in which β-lactamase levels may reflect pre-existing pools and post-transcriptional control rather than acute induction. These observations suggest an alternative, isolate-specific survival strategy that prioritizes drug exclusion over enzymatic inactivation, a hypothesis that warrants functional testing. This strain-specific synergy underscores the need for genotype-guided proteomics to predict resistance phenotypes, as efflux contributions can vary widely across clinical isolates. In multidrug-resistant enterobacteria, resistance, nodulation, and cell division (RND) efflux systems have been shown to synergize with other resistance mechanisms, though their overall impact remains limited.^33^ Our results indicate that different RND systems are expressed at higher levels, likely with an additive effect, thereby increasing resistance to Meropenem.

The primary COG categories in multidrug-resistant *E. coli* isolates that were genomically described include carbohydrate transport and metabolism (G), amino acid transport and metabolism (E), transcription (K), cell wall/membrane/envelope biogenesis (M), inorganic ion transport and metabolism (P), and general function prediction only (R).^34,35^ In this study, under antibiotic pressure, the overrepresented COG categories matched those described in the literature: cell wall/membrane/envelope biogenesis (M) and inorganic ion transport and metabolism (P), which include many proteins in multidrug-resistant *E. coli* that influence bacterial cell permeability.^28,35^ Additionally, signal transduction mechanisms (T), intracellular trafficking, secretion, vesicular transport (U), and defense mechanisms (V) were more common among proteins with higher expression. The overexpression of two-component systems in *E. coli* has been shown to increase antibiotic resistance.^36,37^ A typical example is the ArcAB system; other *Enterobacteriaceae* include CpxAR, PhoPQ, and BaeSR, which have been characterized and are also important in antibiotic resistance.^38,39^ Future global proteomics experiments in ArcAB- or CpxAR-knockout strains could elucidate the causal mechanisms underlying two-component-mediated efflux and metabolic adaptation.

Because *E. coli* CCUG 70745 contains several plasmids carrying antibiotic resistance genes, it may also harbor additional genes involved in intracellular trafficking, secretion, and vesicular transport (U). Secretion systems and flagellar proteins are included in this group, and this category is prominent among broad-host-range plasmids.^40^ Additionally, as shown by Kim et al. (2018), the production of outer membrane vesicles by antibiotic-resistant *E. coli* protects a susceptible strain from various beta-lactams, as these vesicles may contain proteins that degrade beta-lactams or confer protection against them.^41,42^ In this study, most of the upregulated proteins in the 192 μg/mL condition were membrane-associated, and the cell wall compartment was also significantly overrepresented, suggesting a restructuring of the cell wall. Interestingly, many of the proteins showing higher expression are also involved in OMV formation. Further experiments are, however, needed to elucidate the mechanisms and relationship between the generation of OMVs in response to Meropenem. Profiling outer membrane vesicles (OMVs) under Meropenem stress may reveal their roles as a novel auxiliary mechanism. These efforts could inform the development of resistance-disrupting adjuvants, ultimately aiding global AMR surveillance and therapy optimization.

Stress induced by Meropenem treatment in *E. coli* activates general stress responses, as expected.^43^ At the higher antibiotic concentration, according to the network analysis, the two-component system proteins exhibited multiple connections to proteins with diverse functions, suggesting they may sense changes and regulate multiple pathways in the bacterial response to Meropenem, including ABC transporters, oxidative phosphorylation, and cell wall proteins. According to Nagar et al. (2016), two-component system proteins are crucial for crosstalk between stress-affected pathways.^43^ Even when none of the response regulators of the two-component signal transduction system for beta-lactam resistance, as explored by Hirakawa et al. (2003), were identified in the present data set, the role of the cluster in resistance remains unclear.^44^ Among the main modules identified in the DEPs, oxidative phosphorylation, a key part of ATP production in the cell, was linked to ABC transporters and proteins involved in the cell division cycle—processes that require high energy. Bactericidal drugs like Meropenem will trigger cell wall remodeling, continuously draining energy from the cell.^45^

Finally, several virulence- and persistence-associated factors, including type VI secretion system components (Hcp, TssH, and TssM), biofilm regulators and cellulose-associated proteins (BssS, BsmA, BcsE, and cellulose synthase), and the outer membrane protease OmpT, showed altered abundance, indicating that Meropenem exposure may also modulate colonization and biofilm-forming potential of this strain. Although not primary resistance factors, modulation of these virulence- and persistence-associated proteins suggests that Meropenem exposure may also reshape the interaction of carbapenem-resistant *E. coli* with host tissues and microbial communities, potentially influencing colonization, biofilm formation, and survival in antibiotic-rich environments.

The enrichment analysis highlighted different metabolic pathways that were downregulated after Meropenem treatment, including metabolism of cofactors and vitamins (KO00670; KO00740; KO00750; KO00760; KO0770; KO00780; and KO00790), amino acid metabolism (KO00270; KO00300; and KO00480), carbohydrate metabolism (KO00030; KO00051; and KO00052), nucleotide metabolism (KO00240), and energy metabolism (KO00710). Meanwhile, there was higher expression of glycan biosynthesis and metabolism (KO00540 and KO00550), membrane transport pathways (KO02010 and KO03070), drug resistance (KO01503 and KO01501), and biofilm formation. All the processes mentioned require energy from the cell; therefore, in response to this energy demand, oxidative phosphorylation was increased. The increase in the number of proteins involved in thiamine metabolism indicates the importance of the vitamin for bacterial growth; for example, its absence impairs *E. coli* growth.^46,47^ Moreover, thiamine competitors that bind to thiamine-binding sites on enzymes involved in essential metabolic pathways, such as carbohydrate metabolism, will affect bacterial growth and increase antibiotic susceptibility.^46,48^ Similarly, biotin biosynthesis is crucial for establishing infection in various bacterial species;^49^ however, the present data set indicates lower expression of the biotin pathway in CCUG 70745, raising questions about the strain’s infectious capacity under antibiotic stress.

Vitamins, specifically riboflavin, have been shown to inhibit growth in other pathogenic bacteria.^50^ Taken together, the enrichment analysis suggests that it might decrease the metabolism of cofactors and vitamins, which are vital for enzymes involved in other essential metabolic pathways, such as carbohydrate metabolism and nucleotide and protein synthesis, potentially shifting the phenotype to a more stringent state, a hypothesis that requires further investigation and confirmation.

Through various analyses, the data indicate that the acute response of *E. coli* CCUG 70745 to Meropenem is dominated by a coordinated, isolate-specific survival strategy of drug exclusion and envelope reinforcement: rapid upregulation of multidrug efflux (RND systems including AcrAB-TolC and AcrEF) and cell-envelope/peptidoglycan remodeling, energetically supported by increased oxidative phosphorylation and ABC-transporter activity, and associated with abundance changes in envelope- and metal-sensing two-component systems. Secondary, auxiliary features include c-di-GMP-linked surface and biofilm-associated changes, while growth-associated biosynthesis (ribosomes, replication, cofactor, and vitamin biosynthesis) is concurrently downregulated, indicating a growth-versus-defense trade-off. Follow-up studies targeting different two-component systems are needed to better understand their roles in *E. coli* resistance mechanisms. The cell outer membrane, bacterial cell wall, and cell division proteins are highly active. The defense mechanisms that provide resistance in the cells were also significantly upregulated. These processes involve a reduction in the metabolism of carbohydrates, nucleotides, cofactors, and vitamins. The proteome-wide metabolic shifts, particularly the dose-dependent increase in the expression of oxidative phosphorylation and ABC transporters, highlight potential vulnerabilities in CREC that could be exploited for novel therapeutic interventions. For instance, adjunct therapies targeting these pathways, such as inhibitors of energy metabolism or supplements to cofactor biosynthesis, might synergize with carbapenems to overcome efflux-mediated resistance, thereby reducing reliance on last-resort antibiotics. Such approaches align with emerging proteotyping strategies for real-time diagnostics, enabling personalized treatment in clinical settings, where multidrug-resistant infections predominate (Figure 6).

**Figure 6:**
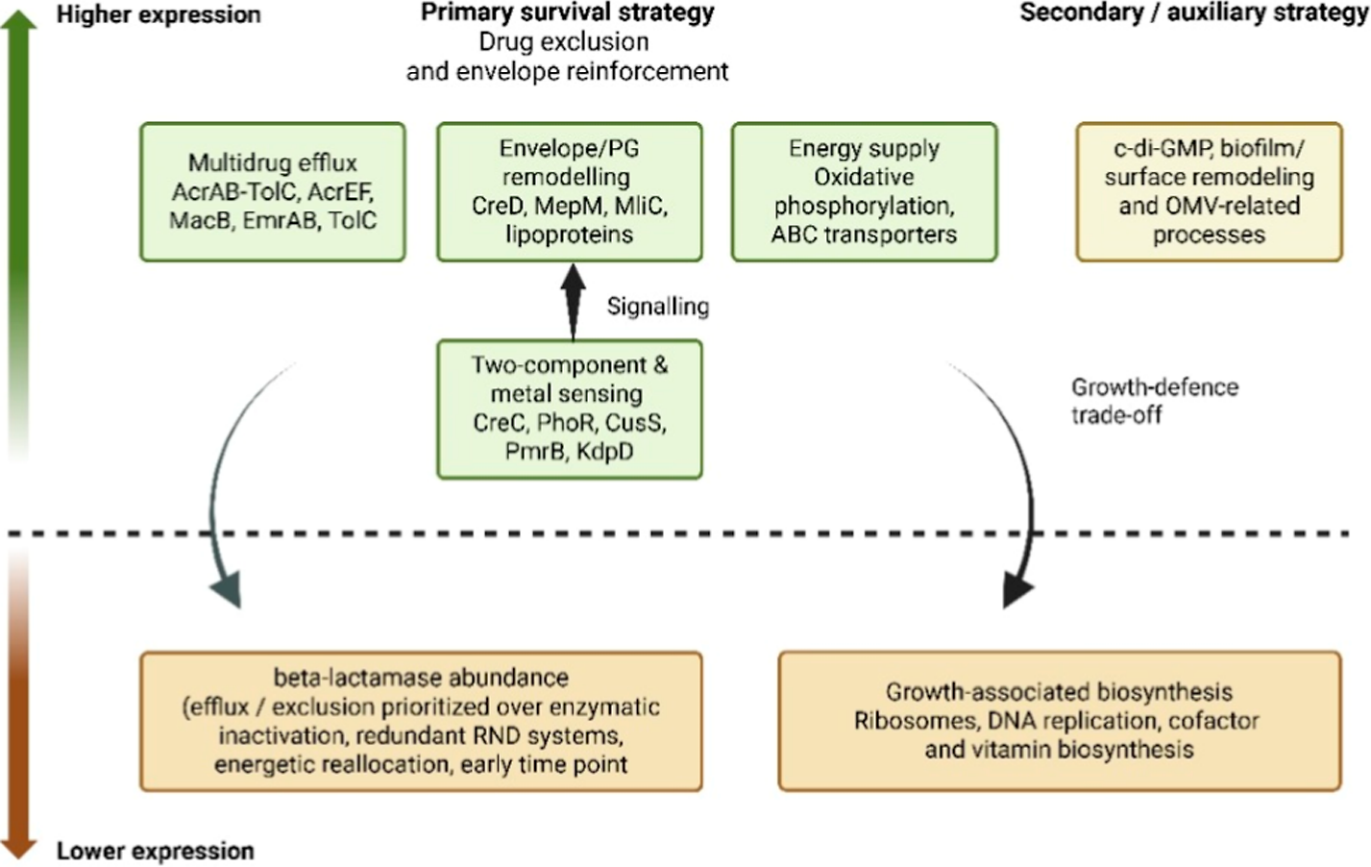
Proposed model of the dose-dependent adaptive response of carbapenem-resistant *E. coli* CCUG 70745 to Meropenem exposure. The primary survival strategy (center) combines drug exclusion and envelope reinforcement: upregulated multidrug efflux (AcrAB-TolC, AcrEF, MacB, EmrAB, and TolC), envelope and peptidoglycan remodeling (CreD, MepM, MliC, and lipoproteins), and energy supply via oxidative phosphorylation and ABC transporters, coordinated by envelope- and metal-sensing two-component signaling (CreC, PhoR, CusS, PmrB, and KdpD). Secondary, auxiliary processes (right) include c-di-GMP-linked surface and biofilm remodeling and OMV-related processes. Concurrently, growth-associated biosynthesis (ribosomes, DNA replication, and cofactor and vitamin biosynthesis) and β-lactamase abundance are downregulated, with efflux and exclusion prioritized over enzymatic inactivation at this early time point, illustrating a growth-versus-defense trade-off. Upward (green) and downward (brown) arrows denote higher and lower relative protein abundance, respectively. Created in BioRender. Karlsson, R. (2026) https://BioRender.com/w46944r.

## Supplementary Material





## Data Availability

The MS proteomics raw data and the results from the search in Proteome Discoverer have been deposited in the ProteomeXchange Consortium (http://www.proteomexchange.org/) via PRIDE^51^ with the data set identifier PXD067730.

## Notes

Detailed methods for genomic DNA isolation and whole-genome sequencing of *E. coli* CCUG 70745, including enzymatic lysis, library preparation, *de novo* assembly (SPAdes), quality assessment (FastQC, QUAST), and strain-identity confirmation against Johnning et al. (2018) by average nucleotide identity (ANIb) and *in silico* DNA–DNA hybridization (GGDC) and growth of *E. coli* strain CCUG 70745 under Meropenem exposure (PDF)
Antibiotic susceptibility profile of *E. coli* strain CCUG 70745 determined by the broth-dilution method, listing antibiotic, abbreviation, class, MIC (μg/mL), and interpretation; complete list of proteins identified and quantified in *E. coli* strain CCUG 70745 after 15 min of Meropenem exposure and in the controls, with normalized abundances, fold changes, *p*-values, and FDR; differentially expressed proteins (DEPs) at 128 μg/mL and 192 μg/mL of Meropenem, including protein description, fold change, *p*-value, FDR, expression direction, COG category, GO terms, and subcellular localization; separate sheets list the proteins with the greatest fold changes at each dose; soft (fuzzy) clustering of the quantified proteins of *E. coli* strain CCUG 70745 under Meropenem exposure, obtained with Mfuzz, with log^2^ fold changes, *p*-values, cluster assignment, and membership values; differentially expressed antibiotic-resistance proteins identified according to the Comprehensive Antibiotic Resistance Database (CARD), with fold change, log^2^ fold change, *p*-value, and FDR at 128 μg/mL and 192 μg/mL; overrepresentation of Clusters of Orthologous Groups (COG) categories among the differentially expressed proteins of *E. coli* strain CCUG 70745 under Meropenem exposure, assessed by Fisher’s exact test (*p*-value, odds ratio, and adjusted *p*-value), for up- and downregulated proteins at each dose; lists of differentially expressed proteins of *E. coli* strain CCUG 70745 under Meropenem exposure used as input for the protein–protein interaction analysis in the STRING database; gene set enrichment analysis (GSEA) of Gene Ontology (GO) terms for the quantified proteins of *E. coli* strain CCUG 70745 under Meropenem exposure, with GO ID, GO name, category, set size, enrichment score (ES), normalized enrichment score (NES), and nominal and FWER *p*-values at 128 μg/mL and 192 μg/mL; and gene set enrichment analysis (GSEA) of KEGG pathways for the quantified proteins of *E. coli* strain CCUG 70745 under Meropenem exposure, with pathway ID, name, set size, ES, NES, and nominal and FWER *p*-values at 128 μg/mL and 192 μg/mL (XLSX)

## Abbreviations

AMR: (antimicrobial resistance)
CAMP: (cationic antimicrobial peptide)
COG: (cluster of orthologous groups)
CREC: (carbapenem-resistant *E. coli*)
DEPs: (differentially expressed proteins)
ESBL: (extended-spectrum beta-lactamase)
FC: (fold-change)
FDR: (false discovery rate)
GO: (gene ontology)
GSEA: (gene set enrichment analysis)
LC-MS/MS: (liquid chromatography-tandem mass spectrometry)
MIC: (minimum inhibitory concentration)
OD: (optical density)
TMT: (tandem mass tag)
PCA: (principal component analysis).
