# Supplementary material for: Proteome Remodeling of a Carbapenem-Resistant Escherichia coli Strain upon Meropenem Exposure
Source: J Proteome Res. 2026 Jul 14;25(8):4276–93. doi: 10.1021/acs.jproteome.6c00342 (PMC13459554; doi:10.1021/acs.jproteome.6c00342)
Supplement: Supplementary file 1 [file pr6c00342_si_001.pdf]

Supporting information

for

Proteome-Remodelling of a Carbapenem-Resistant *Escherichia coli* strain upon Meropenem Exposure

Leonarda Acha Alarcon[1,2], Daniel Jaén-Luchoro[2,3,4], Francisco Salvà-Serra[1,2,3,4,5], Edward R.B. Moore[1,2,3,4], Ivan Mijakovic[6], Roger Karlsson[1,2,3,7,*]

1. Department of Infectious Diseases, Institute for Biomedicine, Sahlgrenska Academy of the University of Gothenburg, 41346 Gothenburg, Sweden

2. Centre for Antibiotic Resistance Research (CARe), University of Gothenburg, 41346 Gothenburg, Sweden

3. Department of Clinical Microbiology, Sahlgrenska University Hospital, Region Västra Götaland, 41346 Gothenburg, Sweden

4. Culture Collection University of Gothenburg (CCUG), Sahlgrenska Academy of the University of Gothenburg, 41346 Gothenburg, Sweden

5. Unit of Medical Technology and Biological Function, Department of Medical Technology and Diagnostics, RISE Research Institutes of Sweden, 43153 Gothenburg, Sweden

6. Systems Biology, Life Sciences, Chalmers University of Technology, 41296 Gothenburg, Sweden

7. Nanoxis Consulting AB, 40016 Gothenburg, Sweden

* Correspondence: roger.karlsson@gu.se

Table of Contents

Supplementary Material 1. Detailed methods for genomic DNA isolation and whole-genome sequencing of *E. coli* CCUG 70745 (PDF)........S-3

Figure S1. Growth of *E. coli* strain CCUG 70745 under meropenem exposure (PDF)........S-4

Supplementary Tables (Microsoft Excel, XLSX) — supplied as separate workbook files

Table S1. Antibiotic susceptibility profile of *E. coli* CCUG 70745 (XLSX)........S-5

Table S2. Complete list of proteins identified and quantified (XLSX)........S-5

Table S3. Differentially expressed proteins (DEPs) at 128 and 192 µg/mL meropenem (XLSX)........S-5

Table S4. Soft (fuzzy) clustering of quantified proteins, Mfuzz (XLSX)........S-5

Table S5. Differentially expressed antibiotic-resistance proteins, CARD (XLSX)........S-5

Table S6. COG category overrepresentation among DEPs (XLSX)........S-5

Table S7. DEP input lists for STRING protein–protein interaction analysis (XLSX)........S-5

Table S8. GSEA of Gene Ontology (GO) terms (XLSX)........S-5

Table S9. GSEA of KEGG pathways (XLSX)........S-5

Supplementary Material 1. Detailed methods for genomic DNA isolation and whole-genome sequencing of ***E. coli* CCUG 70745**

The DNA was isolated using a modification of a previously described protocol (13, 14) with minimal modifications. The enzymatic lysis was performed with 40 µl of Proteinase K (20 mg/ml; Ref. 19131; Qiagen; MD; US) in the presence of sodium dodecyl sulphate (SDS; 25%; Substrate Unit, Department of Clinical Microbiology, Sahlgrenska University Hospital; GBG; SE) at 56 °C for 30 minutes.

Furthermore, the genome was sequenced at Eurofins Genomics (Konstanz, Germany) according to the NGS strategy described elsewhere (15). Pair-end reads were obtained and the quality of the raw reads was assessed using FastQC v.0.11.3 (16). *De novo* assembly was performed using SPAdes v.3.13 (17), and the assembly was evaluated using the Quality Assessment Tool for Genome Assemblies (QUAST) v.4.5 (18). The draft genome was compared to the one previously described by Johnning et al. (2018) using Average Nucleotide Identity (ANIb) and *in silico* DNA–DNA hybridization (GGDC) (20), to confirm the strain's identity.

Figure S1.

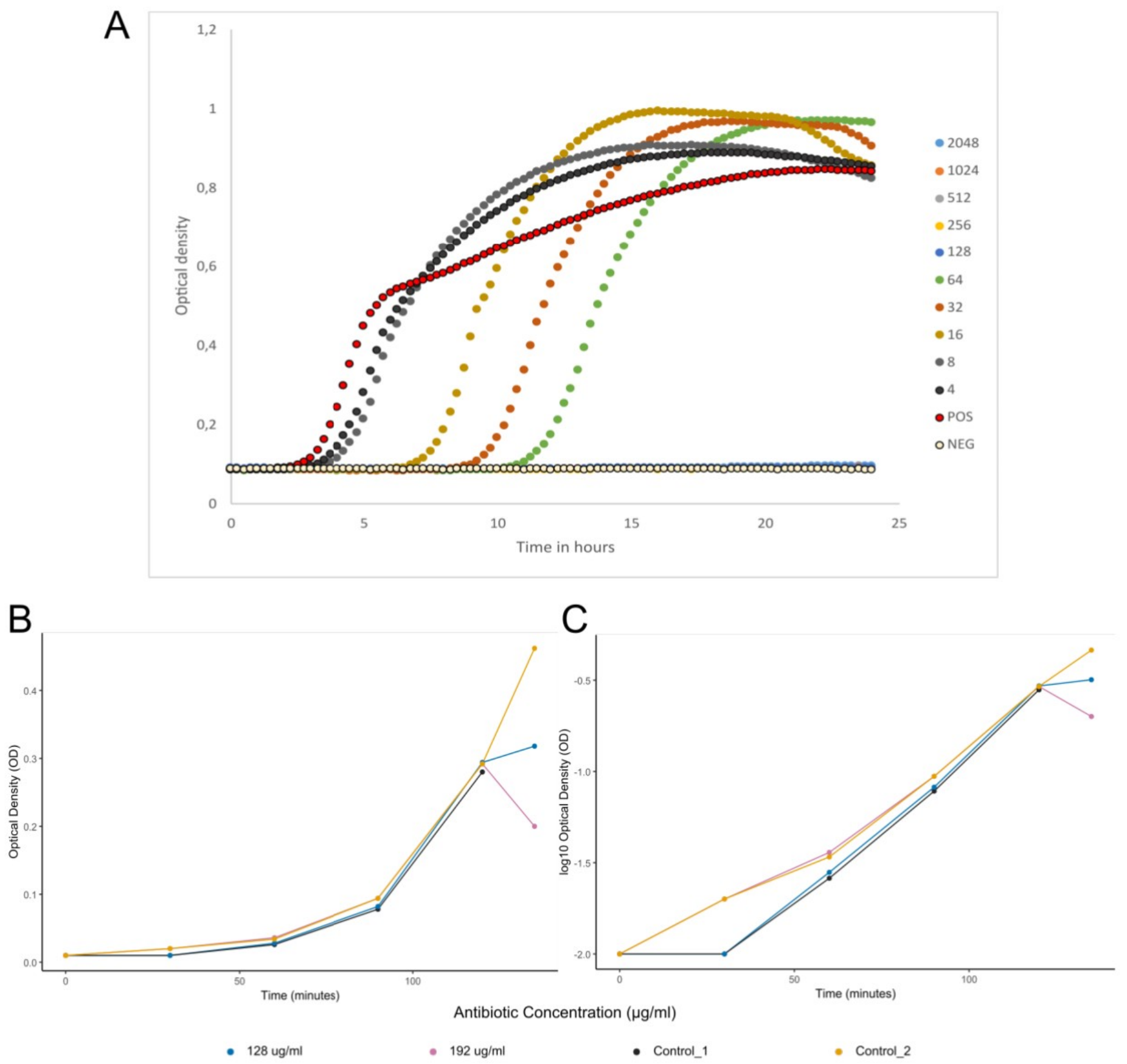

Supplemental Fig. 1. Growth of the strain *E. coli* CCUG 70745 under meropenem exposure.(A) Meropenem Minimal Inhibitory Concentration (MIC) determination by broth dilution method. (B) Growth curves of the proteomics experiment. In the x-axis time in minutes, in the y-axis Optical Density OD at 600 nm of wave lenght. (C) Log10 of the growth curves from the proteomics experiment. In the x-axis time in minutes, in the y-axis log10 of the optical density. The black color corresponds to the control 1, in yellow is represented control 2, light blue is for the culture exposed to 128 µg/ml de meropenem and the pink line represent the cultures at the meropenem concentration of 192 µg/ml.

Supplementary Tables (Microsoft Excel)

The following supplementary tables are provided as separate Microsoft Excel (XLSX) workbook files accompanying this Supporting Information document. The worksheet (tab) names within each workbook are listed for reference.

Table S1. Antibiotic susceptibility profile of *E. coli* strain CCUG 70745 determined by the broth-dilution method, listing antibiotic, abbreviation, class, MIC (µg/mL), and interpretation.

Worksheets: Sheet1

Table S2. Complete list of proteins identified and quantified in *E. coli* strain CCUG 70745 after 15 min of meropenem exposure and in the controls, with normalised abundances, fold changes, p-values, and FDR.

Worksheets: A; B

Table S3. Differentially expressed proteins (DEPs) at 128 µg/mL and 192 µg/mL of meropenem, including protein description, fold change, p-value, FDR, expression direction, COG category, GO terms, and subcellular localisation; separate sheets list the proteins with the greatest fold changes at each dose.

Worksheets: DEPs_128; DEPs_192; greatest_FC_128; greatest_FC_192

Table S4. Soft (fuzzy) clustering of the quantified proteins under meropenem exposure (Mfuzz), with log^2 fold changes, p-values, cluster assignment, and membership values.

Worksheets: cluster_1; cluster_2; cluster_3; cluster_4

Table S5. Differentially expressed antibiotic-resistance proteins identified according to the Comprehensive Antibiotic Resistance Database (CARD), with fold change, log^2 fold change, p-value, and FDR at 128 µg/mL and 192 µg/mL.

Worksheets: Sheet1

Table S6. Overrepresentation of Clusters of Orthologous Groups (COG) categories among the DEPs, assessed by Fisher’s exact test (p-value, odds ratio, adjusted p-value), for up- and down-regulated proteins at each dose.

Worksheets: COG_128_up; COG_128_down; DEPs_192_up; DEPs_192_down

Table S7. Lists of DEPs used as input for the protein–protein interaction analysis in the STRING database.

Worksheets: DEPs_128; DEPs_192

Table S8. Gene set enrichment analysis (GSEA) of Gene Ontology (GO) terms for the quantified proteins, with GO ID, GO name, category, set size, ES, NES, and nominal and FWER p-values at 128 µg/mL and 192 µg/mL.

Worksheets: GSEA_GO_128; GSEA_GO_192

Table S9. Gene set enrichment analysis (GSEA) of KEGG pathways for the quantified proteins, with pathway ID, name, set size, ES, NES, and nominal and FWER p-values at 128 µg/mL and 192 µg/mL.

Worksheets: GSEA_KEGG_128; GSEA_KEGG_192
